# Use of Procalcitonin and C-Reactive Protein to Evaluate Vaccine Efficacy against Pneumonia

**DOI:** 10.1371/journal.pmed.0020038

**Published:** 2005-02-22

**Authors:** Shabir A Madhi, Jayvant R Heera, Locadiah Kuwanda, Keith P Klugman

**Affiliations:** **1**National Health Laboratory Service/University of the Witwatersrand/Medical Research Council Respiratory and Meningeal Pathogens Research Unit, University of the WitwatersrandJohannesburgSouth Africa; **2**Paediatric Infectious Diseases Research Unit, Wits Health ConsortiumUniversity of the Witwatersrand, JohannesburgSouth Africa; **3**Department of International Health, Rollins School of Public Healthand Division of Infectious Diseases, School of Medicine, Emory University, Atlanta, GeorgiaUnited States of America; Soroka University Medical Center and Ben-Gurion University of the NegevIsrael

## Abstract

**Background:**

Pneumonia remains the leading cause of death in young children. The poor specificity of chest radiographs (CXRs) to diagnose pneumococcal pneumonia may underestimate the efficacy of pneumococcal conjugate vaccine in preventing pneumococcal pneumonia.

**Methods and Findings:**

The efficacy of nine-valent pneumococcal conjugate vaccine among children not infected with HIV (21%; 95% confidence interval, 1%–37%) increased when CXR-confirmed pneumonia was associated with serum C-reactive protein of 120 mg/l (12mg/dl) or more and procalcitonin of 5.0 ng/ml or more (64%; 95% confidence interval, 23%–83%). Similar results were observed in children infected with HIV.

**Conclusion:**

C-reactive protein and procalcitonin improve the specificity of CXR to diagnose pneumococcal pneumonia and may be useful for the future evaluation of the effectiveness of pneumococcal conjugate vaccine in preventing pneumococcal pneumonia.

## Abstract 2

Adding two blood measurements to the standard X-ray exam might lead to more specific diagnoses for pneumococcal pneumonia and allow more accurate estimate of the efficacy of pneumococcal vaccines

## Introduction

While pneumonia remains the leading cause of death in children, the absence of sensitive and specific tools to make an etiological diagnosis is a major limitation to our understanding of the efficacy of vaccines against pneumonia. Blood cultures lack sensitivity, and cultures from lung aspirates may be influenced by delayed presentation, antecedent antibiotic therapy, difficulties finding an accessible site to aspirate, lack of skills in performing the procedure, and the perception of both parents and clinicians that the procedure is too invasive [1,2].

Procalcitonin, at a low threshold (≥0.25 ng/ml), has been shown to be useful in directing the use of antibiotics in adults with pneumonia [3], and a recent meta-analysis concludes that procalcitonin may offer some advantages over C-reactive protein (CRP) for discriminating bacterial from nonbacterial infections [4]. Most studies support the observation that children with bacterial infections have higher levels of CRP and procalcitonin than those with viral infections [4,5,6]. Our recent observation that many children with viral-associated pneumonia have a bacterial super-infection [7], however, suggests that a high level of CRP and procalcitonin may be associated with unrecognised bacterial co-infection in a child with an established viral aetiology for pneumonia. This may also explain why some studies have found procalcitonin and CRP not to be useful in distinguishing between bacterial and “viral” pneumonia [4,8].

Based on the postulate that the most likely chest radiograph (CXR) manifestation of pneumococcal pneumonia is alveolar consolidation, we reported the efficacy of a nine-valent pneumococcal conjugate vaccine (PnCV) in reducing CXR-confirmed pneumonia based on definitions recommended by a World Health Organization working group [9]. The observed reduction in the incidence of CXR-confirmed pneumonia in the intent-to-treat analyses in PnCV recipients not infected with HIV (20%; 95% confidence interval [CI], 2% to 35%) and infected with HIV (13%; 95% CI, −7% to 29%) likely underestimated the reduction in the incidence of pneumococcal pneumonia [10]. The reason for this is that the outcome measure (i.e., CXR-confirmed pneumonia) is not highly specific for pneumococcal pneumonia. Consequently, it is likely that many of the CXR-confirmed pneumonia episodes were not pneumococcal in origin and therefore could not have been prevented by the vaccine under evaluation.

We thus evaluated the usefulness of procalcitonin and CRP to improve the specificity of CXR-confirmed pneumonia as an endpoint in vaccine efficacy trials. This analysis was not a primary objective of the study, and is therefore an hypothesis-generating analysis, which should be tested as an a priori hypothesis in other study settings.

## Methods

The methods of the randomised, double-blind, placebo-controlled trial, the objectives of which were to measure PnCV efficacy against invasive pneumococcal disease and CXR-confirmed pneumonia, have been published [10] (Protocol S1). Briefly stated, the study included 39,836 children, including an estimated 6.47% infected with HIV [7], and was performed in Soweto, South Africa. Children were randomised to receive either a nine-valent PnCV conjugated to CRM_197_ (Wyeth-Lederle Vaccines and Pediatrics, Pearl River, New York, United States) or a placebo at 6, 10, and 14 wk of age. The nine-valent PnCV included serotypes 1, 4, 5, 6B, 9V, 14, 18C, 19F, and 23F (i.e., vaccine serotypes) [10]. We now further measured procalcitonin and CRP levels in serum obtained within 12 h of hospitalisation among children with CXR-confirmed alveolar consolidation in the intent-to-treat analysis. CXRs were requested for any child hospitalised with a clinical diagnosis of lower respiratory tract infection according to the diagnosis of the attending study-physician.

CRP values were determined at the time of admission by immunoturbidometry (717 Automated Analyzer, Boehringer Mannheim/Hitachi, Mannheim, Germany) and were reported in milligrams per liter with a lower threshold set at 3 mg/l or lower. In those children in whom CRP was not measured on admission to hospital, archived serum obtained within 12 h of admission and stored at −70 °C was used for analysis. The archived serum samples were also used for measuring quantitative procalcitonin levels using the LUMItest PCT assay (BRAHMS Diagnostica, Berlin, Germany). The CRP and procalcitonin tests were performed using commercially available assays according to the manufacturers' recommendations at the National Health and Laboratory Services, Johannesburg, South Africa.

### Bacterial Cultures

Blood was cultured for bacterial growth on admission and processed using the BacT/Alert microbial detection system (Organon Teknika, Durham, North Carolina, United States). Isolates of Streptococcus pneumoniae were serotyped using the quellung method at the Respiratory and Meningeal Pathogens Research Unit and the results validated at the Statens Serum Institute in Copenhagen, Denmark [10].

### Statistics

Data were analysed using S TATA version 8.0 (StataCorp, College Station, Texas, United States) and Epi Info version 6.04d (Centers for Disease Control and Prevention, Atlanta, Georgia, United States). Vaccine efficacy was calculated using Epi Info based on the formula: vaccine efficacy (percent) = [(ARU − ARV)/ARU] × 100, where ARU is attack rate in unvaccinated individuals and ARV is attack rate in vaccinated indivduals. The vaccine-attributable reduction in disease (VAR) was estimated by measuring the difference in incidence rate between vaccine and placebo recipients and expressed per 100,000 child years of observation. A *p-*value of 0.05 or lower was considered significant.

All analyses were performed on an intent-to-treat basis, which included the first event of CXR-confirmed pneumonia in any child who had received at least a single dose of study vaccine. Vaccine efficacy calculations were also performed for bacteremic pneumococcal pneumonia that included all first episodes of pneumonia associated with growth of S. pneumoniae of any serotype from blood.

### Ethical Considerations

The efficacy study and subsequent study evaluating the role of procalcitonin and CRP in children with CXR-confirmed pneumonia were approved by the Ethics Committee for Research on Human Subjects, University of the Witwatersrand, South Africa (Protocol S2). Guardians gave informed consent for the collection of serum and its use for diagnostic assays that may improve the diagnosis of pneumonia (Protocol S3).

## Results

In children not infected with HIV with CXR-confirmed pneumonia, procalcitonin and CRP values were available for 132 (78.1%) of 169 vaccine recipients and 167 (78.8%) of 212 placebo recipients (*p =* 0.88). The point estimate of vaccine efficacy in this cohort of children for whom serum was available was 21% (*p =* 0.04; 95% CI 1%–37%), not significantly different from that determined in the original study (20%, *p =* 0.03; 95% CI 3%–35%). At CRP levels of 120 mg/l or more in conjunction with the primary CXR-confirmed outcome measure, the estimate of vaccine efficacy increased to 38% (*p =* 0.05); at procalcitonin levels of 5 ng/ml or more, the estimated efficacy increased to 46% (*p =* 0.04); and if both conditions were met, the estimated efficacy increased to 64% (*p* = 0.006) (Table 1).

**Table 1:**
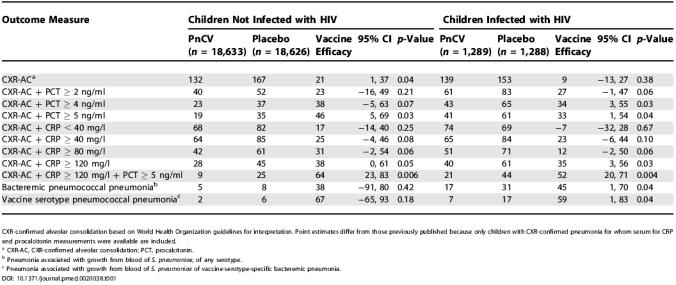
CRPand Procalcitonin Improve the Specificity of CXR to Measure the Efficacy of PnCV in the Prevention of Pneumococcal Pneumonia

CXR-confirmed alveolar consolidation based on World Health Organization guidelines for interpretation. Point estimates differ from those previously published because only children with CXR-confirmed pneumonia for whom serum for CRP and procalcitonin measurements were available are included

^a^ CXR-AC, CXR-confirmed alveolar consolidation; PCT, procalcitonin

^b^ Pneumonia associated with growth from blood of *S. pneumoniae,* of any serotype

^c^ Pneumonia associated with growth from blood of S. pneumoniae of vaccine-serotype-specific bacteremic pneumonia

Among children infected with HIV with CXR-confirmed pneumonia, procalcitonin and CRP values were available for 139 (76.4%) of 182 vaccine recipients and 153 (73.2%) of 209 placebo recipients (*p =* 0.47). Whereas vaccine efficacy was not significant for CXR-confirmed pneumonia (9%; *p =* 0.38), vaccine efficacy was significant when measured against CXR-confirmed pneumonia in conjunction with a CRP level of 120 mg/l or more (35%; *p =* 0.03) or a procalcitonin level of 5 ng/ml or more (33%; *p =* 0.04). As observed in children not infected with HIV, the point efficacy estimate in children infected with HIV with CXR-confirmed pneumonia was greatest if both CRP and procalcitonin were at or above 120 mg/l and 5 ng/ml, respectively (52%; *p =* 0.004; Table 1).

The sensitivity of CXR-confirmed pneumonia plus CRP ≥120 mg/l plus procalcitonin ≥5 ng/ml in detecting vaccine efficacy against pneumococcal pneumonia was analysed by comparing the VAR per 100,000 child years to that observed using an outcome of bacteremic pneumococcal pneumonia. In children not infected with HIV, the combined outcome of CXR-confirmed pneumonia plus CRP ≥120 mg/l plus procalcitonin ≥5 ng/ml (VAR = 37) identified 5.3-fold more cases of pneumonia that were prevented by vaccination than was identified by bacteremic pneumococcal pneumonia (VAR = 7) and 4.1-fold more cases than was identified by vaccine-serotype-specific bacteremic pneumococcal pneumonia (VAR = 9). Similarly, CXR-confirmed pneumonia plus CRP ≥120 mg/l plus procalcitonin ≥5 ng/ml in children infected with HIV (VAR = 772) identified 1.6-fold more cases of pneumonia that were prevented by vaccination than was identified by pneumococcal bacteremic pneumonia (VAR = 483) and 2.2-fold more cases than was identified by vaccine-serotype-specific bacteremic pneumococcal pneumonia (VAR = 344).

Among the children not infected with HIV with CXR-confirmed pneumonia, the likelihood of a difference between placebo and vaccine recipients was significantly increased by the presence of a CRP level of 120 mg/l or more and a procalcitonin level of 5 ng/ml or more (odds ratio = 1.37; 95% CI, 1.09–1.73; *p =* 0.027). This was true also among children infected with HIV (odds ratio = 1.41; 95% CI, 1.14–1.75; *p =* 0.005).

## Discussion

Our results show that the true efficacy of the PnCV against pneumococcal pneumonia may be underestimated if one relies solely on a radiological diagnosis of pneumococcal pneumonia. The lack of specificity of CXRs for inferring bacterial versus non-bacterial etiology of pneumonia has been previously described [11,12]. The specificity of CXR-confirmed pneumonia as an outcome measure of efficacy of PnCV to prevent pneumococcal pneumonia was improved when it was analysed together with indirect evidence suggestive of bacterial infection, namely, elevated CRP and procalcitonin levels. This outcome measure of CXR-confirmed pneumonia plus CRP ≥120 mg/l plus procalcitonin ≥5 ng/ml, while more specific than CXR-confirmed pneumonia alone, will not be 100% accurate because pneumonia associated with other bacteria, including that due to pneumococcal serotypes against which the PnCV has no effect, may well present in a similar manner.

An alternate explanation for our results, rather than that of improved specificity in diagnosing pneumococcal pneumonia, may be that the criteria of CXR-confirmed pneumonia coupled with a CRP level of 120 mg/l or more and a procalcitonin level of 5 ng/ml or more detected more severe disease against which the vaccine may have been more efficacious. Although we are unaware of data to support that CRP and procalcitonin are elevated in those with severe pneumonia, the absence of such data supporting this interpretation does not rule out this possibility.

Importantly, the levels of CRP and procalcitonin used in this report are designed for specificity rather than sensitivity; it would be inappropriate to use the same threshold values of CRP or procalcitonin during the course of routine clinical practice when managing individuals suspected of having bacterial pneumonia. In that instance, it would be more appropriate to use thresholds that have a high sensitivity, to ensure that all children with possible bacterial infection are adequately treated [3,4,8].

Procalcitonin and CRP were equally useful in children infected and not infected with HIV to improve the specificity of the pneumococcal pneumonia efficacy endpoint in the vaccine trial among children with CXR-confirmed pneumonia. This is particularly important, as our initial observation of a non-significant reduction in CXR-confirmed pneumonia was attributed to the complexity of etiology of pneumonia, and thus the even poorer specificity of CXRs for identifying pneumococcal pneumonia in children infected with HIV compared to children not infected with HIV [13,14]. The current study thus indicates that the specificity of CXR-confirmed pneumonia for diagnosing pneumococcal pneumonia may also be improved in children with HIV through the concurrent use of procalcitonin and CRP.

A distinction must be made between measures of vaccine efficacy for which the increased specificity of CXR-confirmed pneumonia plus CRP ≥120 mg/l plus procalcitonin ≥5 ng/ml over CXR-confirmed pneumonia alone is apparent, and studies of the burden of disease prevented (VAR), which are optimised when vaccine sensitivity is maximised. Our data show that the VAR is greater for CXR-confirmed pneumonia plus CRP ≥120 mg/l plus procalcitonin ≥5 ng/ml than that calculated using the very highly specific endpoints of serotype-specific or all-serotype pneumococcal bacteremic pneumonia. The best estimates of VAR will, however, be provided by endpoints that sacrifice some specificity for maximal sensitivity. Such studies include less specific clinical endpoints and are outside of the scope of the current analysis [7].

Our findings have implications for the planning of future studies aimed at evaluating the effectiveness of pneumococcal vaccines against bacterial pneumonia. Our data suggest that the outcome measure of CXR-confirmed pneumonia together with elevated CRP and procalcitonin levels may be more accurate as a surrogate of pneumococcal pneumonia than CXRs on their own. The increased specificity of this endpoint may allow smaller sample sizes for future vaccine efficacy/effectiveness studies. Based on the incidence of CXR-confirmed pneumonia in this report (167 [0.9%] of 18,626), a sample size of 80,058 children were required to detect the 20% reduction in CXR-confirmed pneumonia with 80% power and an alpha of 0.05. The actual power for this outcome in this study was thus only 46.8%. Using the more specific outcome measure of CXR-confirmed pneumonia with a CRP level of 120 mg/l or more and a procalcitonin level of 5 ng/ml or more, the sample size required to detect the observed 64% reduction in outcome was 44,734, i.e., 56% of the sample size required when measuring vaccine efficacy against CXR-confirmed pneumonia alone, and the power of the current study would have been increased from 46.8% to 71.5%.

Our findings therefore suggest that CXR-confirmed pneumonia coupled with serological markers of CRP and procalcitonin is a more specific marker of pneumococcal pneumonia and may therefore provide a closer estimate of the efficacy of the PnCV against pneumococcal pneumonia.

## Supporting Information

Protocol S1 Protocol of the Original Phase 3 Study (250 KB DOC).

Protocol S2 Ethics Committee Letter of Approval (378 KB DOC).

Protocol S3 Informed Consent Form for Original Phase 3 Study (24 KB DOC).

**Patient Summary**

**Background**

Pneumonia is the leading cause of death in children worldwide. Pneumonia can be caused by different bacteria and viruses, and there are no easy diagnostic tests to find out which bacterium or virus has caused the disease in a particular patient. This not only causes problems with prescribing the best treatment, but also makes it hard to evaluate vaccines that might protect against some causes of the disease but not others.

**Why Was This Study Done?**

The researchers involved in this study have evaluated vaccines against a particular bacterium (called *Pneumococcus*) that is the leading cause of pneumonia in children. They have begun to test these vaccines in children, but were looking for more specific ways to distinguish cases of pneumonia caused by this particular bacterium from those caused by other bacteria or viruses.

**What Did the Researchers Do?**

They had previously done a trial for a vaccine that relied on chest X-rays to diagnose pneumonia. They had also collected blood from children who had participated in the trial and had become sick with pneumonia. They now checked those blood samples for two markers that indicate a bacterial infection and re-analyzed the study.

**What Did They Find?**

The vaccine was able to protect children—to some extent—against pneumonia. The vaccine appeared to offer greater protection against pneumonia when the pneumonia was diagnosed by a combination of X-rays and high levels of the two blood markers than when the illness was diagnosed just with a chest X-ray.

**What Does This Mean?**

These results raise the possibility that a combined test (chest X-ray plus two blood markers) is better at assessing whether the pneumonia vaccine works than just a chest X-ray alone.

**What Next?**

Because of the way this study was done—adding a specific analysis to a clinical trial after it was completed, rather than planning to test a hypothesis from the outset—it cannot be considered as proof of the idea tested. The results suggest that it is worth testing whether the combined diagnosis is more specific for pneumococcal pneumonia than chest X-rays alone, but new studies are needed to resolve the issue.

**More Information Online**

The Global Alliance for Vaccines and Immunization (GAVI): http://www.vaccinealliance.org/

GAVI Web page “Call for Intensified Research after Pneumococcus Trial Surprises”: http://www.vaccinealliance.org/Resources_Documents/Immunization_Focus/Download/update.php

World Health Organization (WHO) Web site on vaccines: http://www.who.int/vaccines/

WHO Web page “Pneumococcal Vaccines”: http://www.who.int/vaccines/en/olddocs/pneumococcus.shtml)

## Abbreviations

CI: confidence interval
CRP: C-reactive protein
CXR: chest radiograph
PnCV: pneumococcal conjugate vaccine
VAR: vaccine-attributable reduction in disease

## References

[pmed-0020038-b1] [Anonymous] (1988). Pneumonia in childhood. Lancet.

[pmed-0020038-b2] Vuori-Holopainen E, Peltola H (2001). Reappraisal of lung tap: Review of an old method for better etiologic diagnosis of childhood pneumonia. Clin Infect Dis.

[pmed-0020038-b3] Christ-Crain M, Jaccard-Stolz D, Bingisser R, Gencay MM, Huber PR (2004). Effect of procalcitonin-guided treatment on antibiotic use and outcome in lower respiratory tract infections: Cluster-randomised, single-blinded intervention trial. Lancet.

[pmed-0020038-b4] Simon L, Gauvin F, Amre DK, Saint-Louise P, Lacroix J (2004). Serum procalcitonin and C-reactive protein levels as markers of bacterial infection: A systematic review and meta-analysis. Clin Infect Dis.

[pmed-0020038-b5] Moulin F, Raymond J, Lorrot M, Marc E, Coste J (2001). Procalcitonin in children admitted to hospital with community acquired pneumonia. Arch Dis Child.

[pmed-0020038-b6] Gendrel D, Raymond J, Coste J, Moulin F, Lorrot M (1999). Comparison of procalcitonin with C-reactive protein, interleukin 6 and interferon-alpha for differentiation of bacterial vs. viral infections. Pediatr Infect Dis J.

[pmed-0020038-b7] Madhi SA, Klugman KP, The Vaccine Trialist Group (2004). A role for *Streptococcus pneumoniae* in virus-associated pneumonia. Nat Med.

[pmed-0020038-b8] Korppi M, Remes S (2001). Serum procalcitonin in pneumococcal pneumonia in children. Eur Respir J.

[pmed-0020038-b9] World Health Organization Pneumonia Vaccine Trial Investigators' Group (2001). Standardization of interpretation of chest radiographs for the diagnosis of pneumonia in children. Geneva: World Health Organization. Available: http://www.who.int/vaccine_research/documents/en/pneumonia_children.pdf. http://www.who.int/vaccine_research/documents/en/pneumonia_children.pdf.

[pmed-0020038-b10] Klugman KP, Madhi SA, Huebner RE, Kohberger R, Mbelle N (2003). A trial of a 9-valent pneumococcal conjugate vaccine in children with and those without HIV infection. N Engl J Med.

[pmed-0020038-b11] Courtoy I, Lande AE, Turner RB (1989). Accuracy of radiographic differentiation of bacterial from nonbacterial pneumonia. Clin Pediatr (Phila).

[pmed-0020038-b12] Friis B, Eiken M, Hornsleth A, Jensen A (1990). Chest X-ray appearances in pneumonia and bronchiolitis. Correlation to virological diagnosis and secretory bacterial findings. Acta Paediatr Scand.

[pmed-0020038-b13] Madhi SA, Cumin E, Klugman KP (2002). Defining the potential impact of conjugate bacterial polysaccharide-protein vaccines in reducing the burden of pneumonia in human immunodeficiency virus type 1-infected and -uninfected children. Pediatr Infect Dis J.

[pmed-0020038-b14] Madhi SA, Cutland C, Ismail K, O'Reilly C, Mancha A (2002). Ineffectiveness of trimethoprim-sulfamethoxazole prophylaxis and the importance of bacterial and viral coinfections in African children with Pneumocystis carinii pneumonia. Clin Infect Dis.

