# Supplementary material for: Use of Procalcitonin and C-Reactive Protein to Evaluate Vaccine Efficacy against Pneumonia
Source: PLoS Med. 2005 Feb 22;2(2):e38. doi: 10.1371/journal.pmed.0020038 (PMC549587; doi:10.1371/journal.pmed.0020038)
Supplement: Protocol S1 — (250 KB DOC). [file pmed.0020038.sd001.doc]

**CONFIDENTIAL**

**Double-blind, Randomized Trial of Nonavalent Pneumococcal Conjugate Vaccine to Reduce the Incidence of Invasive Pneumococcal Disease and Pneumonia Requiring Hospitalization in Infants**

**Principal Investigator:**

**Keith P. Klugman, MBBCh, MMed (Microbiol), PhD, FCPath (SA), DTM&H, MRC Path (London), FRSSAfr**

**Medical Research Council/ South African Institute for Medical Research**/**University of the Witwatersrand**

**Pneumococcal Diseases Research Unit,**

**Johannesburg, South Africa**

**Protocol No: WLVP D124-P502**

**Issue Date:**

**Vaccine Supplier:**

**Wyeth-Lederle Vaccines and Pediatrics**

**211 Bailey Road**

**West Henrietta, NY 14586**

**Co-Investigators:**

**Prof. J.M. Pettifor**

**Head, Department of Pediatrics,**

**Chris Hani Baragwanath Hospital**

**Dr. Shabir Mahdi**

**Consultant pediatrician**

**MRC/SAIMR/WITS Pneumococcal Diseases Research Unit**

**Dr. Nontombi Mbelle**

**Consultant microbiologist**

**MRC/SAIMR/WITS Pneumococcal Diseases Research Unit**

**Dr. Robin Huebner**

**Infectious Disease Epidemiologist**

**MRC/SAIMR/WITS Pneumococcal Diseases Research Unit**

Protocol Summary 1

I. Background and rationale 4

A. Invasive pneumococcal disease 4

B. Invasive pneumococcal disease in Southern Africa 4

C. Acute lower respiratory tract infections as a global problem .5

D. Acute lower respiratory tract infections in South Africa .6

E. The HIV epidemic in South Africa .6

F. Pneumococcal conjugate vaccine trials -potential for intervention .7

G. Pneumococcal serotype\serogroup distribution and potential vaccine coverage .8

H. Description of pneumonia surveillance study .9

II. Study objectives 10

A. Primary objectives of the study 10

B. Secondary objectives of the study 10

III. Study site 11

IV. Subject eligibility 12

A. Inclusion Criteria 12

B. Exclusion Criteria 12

C. Conditions which might require delaying administration of vaccination 13

D. Contraindications for subsequent doses of the study vaccine 13

V. Trial design 13

A. Enrolment 13

B. Randomization 13

C. Blinding 14

D. Treatment period 14

E. Control group 14

F. Follow-up 15

1. Vaccine Safety 15

2. Vaccine Immunogenicity 15

3. Vaccine Efficacy 16

G. Termination of follow-up for outcome surveillance 16

VI. Conduct of the study 16

A. Enrolment 16

B. Administration of initial and subsequent vaccinations 17

C. Immunogenicity study 19

D. Assessment of vaccine safety 19

E. Assessment of vaccine efficacy 20

1. Identification of study participants admitted to hospital with possible infectious disease etiology 20

2. Investigation of hospitalized study-patients 21

a. Routine investigations 21

b. Additional tests that are specific to the study 21

c. Microbiology methods 22

VII. Vaccines and vaccine management 22

A. Nonavalent pneumococcal conjugate vaccine 22

B. ‘Control’ vaccine 22

C. Other vaccines administered at 6, 10 and 14 weeks 22

D. Management of study vaccines 22

VIII. Case definition 23

A. Determination of efficacy against vaccine serotype invasive pneumococcal disease in HIV-uninfected children 23

B. Determination of efficacy against pneumonia in HIV-uninfected children 23

C. Clinical definition of cases 24

1. Hospitalized pneumonia 24

2. Graded ARI definitions 24

a. Acute lower respiratory tract infection - non severe . . 24

b. Severe pneumonia 25

c. Severe acute lower respiratory tract infection . . 25

d. Very severe pneumonia 25

3. Pneumococcal pneumonia 25

4. Pneumococcal meningitis 25

5. Other invasive pneumococcal disease 25

a. Pneumococcal peritonitis 26

b. Pneumococcal septic arthritis 26

c. Pneumococcal cellulitis 26

d. Pneumococcal septic shock without a focus 26

IX. Sample size and plan for analyses 26

A. Primary outcome variables 26

B. Statistical analysis for the primary outcome variables 27

C. Secondary efficacy outcome variables and statistical analyses 29

D. Other analyses 30

E. HIV-positive population 30

F. Estimated study mortality 31

G. Interim analysis 31

X. Adverse reactions 31

XI. Data management and data quality control 32

XII. Study monitoring 32

A. Safety 33

B. Progress of the study 33

C. Verification of data 33

XIII. Study advisory committees 34

A. International Advisory Committee (IAC) 34

B. Safety Monitoring Committee (SMC) 35

XIV. Rules for unblinding the study 37

XV. Target dates 37

XVI. Study management 37

A. Monitoring 37

B. Protocol Revisions 38

C. Data management 38

D. Publications 38

E. Materials 38

F. Indemnification 38

XVII. References 39

Appendix 1: Data of most common infection-related diagnosis in 1995 compared to 1993 and 1994 42

Appendix 2: Association between HIV and other common bacterial infections 43

Appendix 3: Immunization clinics in Soweto and Orange Farm 44

Appendix 4a: Subject information sheet for study participation 45

Appendix 4b: Subject information sheet for immunogenicity study 47

Appendix 5: Manufacturer’s package insert - TETRAMUNE® 48

**Protocol Summary**

This randomized, double-blind, placebo-controlled study will evaluate the clinical efficacy of nonavalent (serotypes 1, 4, 5, 6B, 9V, 14, 18C, 19F, 23F) conjugate pneumococcal vaccine in infants in Soweto, Johannesburg, South Africa. Vaccine efficacy will be determined by comparison to a control group of children receiving a placebo identical in appearance to the pneumococcal conjugate vaccine. Both the vaccine and placebo are manufactured by Wyeth-Lederle Vaccines and Pediatrics, USA (WLVP). The conjugate vaccine contains, in a 0.5 ml dose, 2 μg each of polysaccharides belonging to serotypes 1, 4,5, 9V, 14, 19F and 23F; 2μg of oligosaccharide of serotype 18C; and 4 μg of 6B polysaccharide. All the polysaccharides are independently coupled to a total of approximately 20 μg of CRM 197, a non-toxic mutant of diphtheria toxoid, that acts as a protein carrier. Patients in the control group will receive a placebo containing sucrose, the stabilizing material for the conjugate pneumococcal vaccine. As a benefit to the placebo group, all children will receive *Haemphilus influenzae* type b conjugate vaccine. This vaccine is licensed in South Africa but is not yet available in the EPI Programme.

The primary objectives of the study are:

1. To determine the protective efficacy of the nonavalent conjugate pneumococcal vaccine against invasive pneumococcal disease due to serotypes in the vaccine, occurring ≥14 days following immunization, in HIV-uninfected (HIV ELISA negative or HIV ELISA positive/HIV PCR negative) children who have received three doses of the vaccine per protocol. Invasive pneumococcal disease will be defined as isolation of *S. pneumoniae* from any normally sterile body fluid, including blood, cerebrospinal fluid, pleural fluid, ascitic fluid, and synovial fluid.

2. To determine the clinical efficacy of the conjugate nonavalent pneumococcal vaccine in reducing radiologically-confirmed pneumonia requiring hospitalization in HIV-uninfected children who have received three doses of the vaccine per protocol.

B. The secondary objectives of the study are:

For HIV-uninfected children:

1. To evaluate the efficacy of the pneumococcal conjugate vaccine on the incidence of invasive pneumococcal disease due to all pneumococcal serotypes in children who have received three doses of study vaccine per protocol.

2. To evaluate the efficacy of two or more doses of the pneumococcal conjugate vaccine against vaccine serotype-specific invasive disease and pneumonia requiring hospitalization.

3 To determine the protective efficacy of the pneumococcal conjugate vaccine against vaccine serotype invasive disease in an ‘intent-to-treat’ analysis, i.e., analysis including all randomized children receiving at least one dose of study vaccine.

4. To further assess the safety and immunogenicity of the conjugate pneumococcal vaccine.

5. To derive a population-based serological correlate of protection.

For HIV-infected children (HIV ELISA positive and confirmed positive by PCR):

1. To evaluate the efficacy of pneumococcal conjugate vaccine on invasive pneumococcal disease, as previously defined, caused by vaccine serotypes in children receiving three doses of study vaccine per protocol.

2. To evaluate the efficacy of pneumococcal conjugate vaccine on the incidence of invasive pneumococcal disease caused by all serotypes in children who have received three doses of study vaccine per protocol.

3. To evaluate the efficacy of pneumococcal conjugate vaccine on the incidence of pneumonia in hospitalized children who have received three doses of study vaccine per protocol.

4. To evaluate the efficacy of two or more doses of the pneumococcal conjugate vaccine on the incidence of vaccine serotype-specific invasive disease and pneumonia requiring hospitalization in children.

5. To assess the impact of pneumococcal vaccination on pneumonia-related and all cause mortality and time to death of hospitalized HIV-infected children.

All children presenting to immunization clinics in Soweto or Orange Farm, an informal settlement just outside of Soweto, for routine immunization at approximately 6 weeks of age will be evaluated for study participation. A brief medical history and physical examination will ascertain whether the child meets all inclusion criteria and none of the exclusion criteria. It is anticipated that the entire birth cohort over a two-year period (approximately 48,000 children) will be recruited into the study; it is estimated that there will be a minimum of 28 cases of vaccine-serotype pneumococcal disease in HIV-uninfected children.

Subjects will be randomized at each recruitment site in equal numbers to receive either the pneumococcal conjugate vaccine or placebo; there will be ten vaccine groups, designated 0-9. Randomization schemes for each recruitment site will be provided prior to the start of the study by WLVP. Each study subject will receive three injections of either pneumococcal conjugate vaccine or placebo, administered concurrently with TETRAMUNE® (DTPHib), OPV, and Hep B. The injections will be administered at approximately 6, 10, and 14 weeks of age, with a minimum interval of 3 weeks between each dose and all three doses being completed by 9 months of age, as per protocol. Vaccine safety will be evaluated by monitoring each child for approximately 15 minutes after vaccination; the parent/guardian will also be asked to observe for untoward effects which might occur within one week of vaccination. Study pediatricians will conduct daily surveillance for hospitalization of study participants. The frequency of adverse events following vaccination will be assessed for both the placebo and treatment groups.

Vaccine efficacy will be defined as the number of cases of culture-confirmed invasive pneumococcal disease or pneumonia requiring hospitalization, in the HIV-negative group receiving the pneumococcal conjugate vaccine compared to the number of cases occurring in the placebo group. The study pediatricians will conduct daily surveillance, including ward visits, at Chris Hani Baragwanath Hospital, the referral hospital for Soweto and surrounding area. They will also obtain daily lists from the hospital microbiology laboratories of patients with specimens positive for *S. pneumoniae* confirmed pneumococcal disease. HIV testing will be performed in an anonymous manner for all children with study outcomes who are not tested for HIV during hospitalization. Those outcome cases with no HIV test results will be excluded from the efficacy analyses but will be included in the “intent to treat” analysis.

No formal interim analysis of the efficacy data is planned and no early termination of the study due to early evidence of efficacy is planned. Once 28 HIV-negative “primary objective 1" outcome cases have been accrued, enrolment into the study will stop. The International Advisory Committee for the study will then meet to discuss whether/how long surveillance for outcome cases should continue. Surveillance will likely continue until 43 total cases have been accrued.

To confirm the immunogenicity of the vaccine used in the study, a subset of 200 children will have blood taken at 18 weeks. The children will be randomly-selected from the pool of all children who have received 3 doses of the vaccine or placebo. Serum antibody responses to the pneumococcal vaccine will be measured by standardized ELISA methods. Anonymous and confidential HIV testing will also be performed to estimate the HIV seroprevalence in the study population. The immunogenicity study will be repeated when each additional lot of pneumococcal conjugate vaccine is introduced into the study.

**I. Background and rationale**

**A. Invasive pneumococcal disease**

*Streptococcus pneumoniae* is estimated to cause 1.2 million deaths due to pneumonia and a further 140,000 deaths from meningitis and/or septicemia among children under 5 years of age worldwide[1]. It is the single most important cause of childhood death [2]. In developing countries *S. pneumoniae* is particularly important as an etiological agent of pneumonia and meningitis. While respiratory viruses predominate in industrialised countries, bacteria are the most important etiological agents of severe childhood pneumonia in developing countries [3,4]. The importance of bacteria as a cause of pneumonia is supported by recent data from The Gambia which suggest that 21% of radiographically-confirmed severe pneumonia may be reduced by vaccination with *Haemophilus influenzae* type b conjugate vaccine (Hib) [5]. The potential impact of a vaccine against *S. pneumoniae* is highlighted by the fact that the pneumococcus is a more important respiratory pathogen than *H. influenzae*. Indeed, in The Gambia more than two-thirds of bacterial isolates in children with pneumonia are due to *S. pneumoniae* [3,4]. It is estimated that with the advent of an effective pneumococcal vaccine for children under 2 years of age, deaths from invasive pneumococcal disease could be decreased by 400,000-580,000 [2].

The incidence of invasive pneumococcal disease shows wide geographic variation and is related to socioeconomic status and possibly ethnic backgrounds. Incidences of invasive pneumococcal disease in children under 2 years range from 1195/ 100,000 in Alaskan natives [6], 458/100,000 in The Gambia [7], 145/100,000 in Northern California [8], to 45.3/100,000 in Finland [9] and 26/100,000 in Sweden [10]. The case fatality rate from invasive pneumococcal disease also varies from 3.2% in Alaska to 1.3% in Finland [6,9].

The spectrum of disease is equally variable. In developing countries pneumonia, meningitis and bacteremia without a focus are common [6,7,11], while in industrialised countries mucosal infections, particularly otitis media, are the dominant expression of pneumococcal disease. [9,11]. In addition, the emergence of antimicrobial resistance in the pneumococcus has greatly increased the cost of treatment for otitis media, which is the most important reason for child health care visits in developed countries. In San Francisco 79% of *S. pneumoniae* in children less than 2 years of age were isolated in an outpatient setting [8].

**B. Invasive pneumococcal disease in Southern Africa**

Crewe-Brown et al [12], in a retrospective study from 1993-1995 identified 1012 patients, including 252 children, with positive pneumococcal blood culture isolates at Chris Hani Baragwanath Hospital in Soweto, South Africa. Based on population estimates performed in 1995 [13], this would suggest an overall incidence of pneumococcal disease for all ages of 28\100,000 per year in Soweto. The incidence of a positive pneumococcal blood culture isolate for children under 5 years is estimated at 69\100,000\ year and 160\100,000 in children under 2 years [12].

The incidence of *S. pneumoniae* among HIV-infected children is estimated to be 1114/100,000/year based on 1996 data [Jones et al 1997- manuscript in preparation]. Jones et al also showed that more than 90% of bacteremias occur in children younger than 2 years and that 51% of children with *S. pneumoniae* bacteraemia are HIV-infected.

The incidence of invasive pneumococcal disease is probably higher as the study by Crewe-Brown et al [12] did not include *S. pneumoniae* isolates from other normally sterile body fluid sites and did not include patients from Soweto who may have presented elsewhere for medical attention. In addition it is widely recognised that only 20-30% of children with pneumonia have a positive bacterial isolate on blood culture [3,4] and the pneumococcus is not isolated in all children with *S. pneumoniae* meningitis. The incidence of invasive pneumococcal disease can be estimated to be approximately 5-fold higher than the incidence reflected by positive *S. pneumoniae* blood isolates. Based on the latter assumptions, the incidence of invasive pneumococcal disease among children under 5 years is approximately 340\100,000\year and 765/100,000 in children under 2 years of age.

An earlier study of nonmeningitic invasive pneumococcal bacteremia at Chris Hani Baragwanath Hospital found that 75% of the patients had pneumonia, 20% had bacteremia without a focus and 5% had peritonitis [14]. The case fatality rate for pneumococcal pneumonia was 12%. In that study, 32% of all *S. pneumoniae* showed evidence of penicillin resistance; penicillin-resistant pneumococci were more common among HIV-infected children [14]. The study by Crewe-Brown et al also found increased penicillin-resistant pneumococcal disease in HIV-infected compared to uninfected children [12]. Friedland et al demonstrated that multi-drug resistance was present in 8.8% of isolates and that penicillin-resistance was evident among 40% of community-acquired *S. pneumoniae* infections and 94.9% of nosocomial-acquired infection [15].

**C. Acute lower respiratory tract infections as a global problem**

The WHO estimates that, in 1990, 4.3 million of the 12.9 million deaths in children under 5 years of age were due to acute lower respiratory tract infections [16]. More than 90% of these deaths occurred in developing countries, where children less than 5 years of age account for 15% of the population, but contribute 50% of all deaths. Acute respiratory infections are also responsible for significant morbidity in this age group, resulting in 30-50% of the total visits to health centers and 20-40% of all hospital admissions [17]. Acute respiratory infection as a cause of morbidity and mortality are likely to increase in view of the HIV pandemic. It is estimated that 1 million children are HIV-infected, 80-90% of these children are in sub-Saharan Africa.

**D. Acute lower respiratory tract infections in South Africa**

According to UNICEF data, the national under 5-mortality rate in South Africa is 68\1000 [18]. The total number of deaths in children less than 5 years of age is 85,000. The infant mortality rate for South Africa is estimated at 52\1000 and the annual number of births at 1,247,000 [1].

Based on limited data from the Central Statistical Services of South Africa, Von Schirnding et al [19] reported that between 1980-1985 the under 5 mortality rate from acute lower respiratory tract infection among ‘coloured’ and ‘Caucasian’ children was 981 and 88 per 100,000 children, respectively.

The average number of deaths, in children under 5 years, reported as pneumonia during this period were 93, 861, and 3254 per 100,000 children for ‘Caucasian’, ‘coloured’, and ‘black’ children, respectively. The data for ‘blacks’ are unreliable and probably under represent the true figures. Among the ‘coloured’ children, pneumonia was responsible for 90% of acute respiratory infection related deaths [19].

At Chris Hani Baragwanath Hospital pneumonia is the most important cause of death among hospitalised children (Appendix 1). In 1995 the number of deaths recorded as pneumonia had doubled compared to 1994 and tripled since 1992. Children under 2 years of age accounted for 69.2% of pediatric admissions and 92.3% of all deaths. The increase in morbidity and mortality related to pneumonia is mirrored by an increase in the diagnosis of HIV infection\AIDS [Appendix 1,2]. In 1995 at Chris Hani Baragwanath Hospital, the case fatality rate from pneumonia was 6.29%. The incidence of acute lower respiratory tract infection in Soweto, particularly severe pneumonia, will be prospectively investigated in 1997.

In Zimbabwe, the overall case-fatality-rate from acute lower respiratory tract infections was 15%. The case fatality rate varied from 28% in clinically HIV-infected children compared to 9% in clinically uninfected children. Of further significance, 67% of fatal cases occurred in children under 6 months of age [20].

**E. The HIV epidemic in South Africa**

A recently completed study found that 30% of children admitted to the general pediatric wards were HIV-infected [Dr Meyers-unpublished]. Among women attending antenatal clinics in Gauteng, the province which includes the Johannesburg and Soweto areas, the seroprevalence of HIV in 1995 was 12%. The overall maternal-to-child transmission rate at Chris Hani Baragwanath Hospital is 32%. Using these statistics, it is estimated that at least 4% of all newborns are HIV-infected [Dr. Gray- manuscript in preparation].

The high prevalence of HIV among children has major implications for the epidemiology and management of acute lower respiratory tract infection and invasive pneumococcal disease. The incidence of invasive pneumococcal disease in HIV-infected patients is 100-300 fold greater than the general population [8,21,22]. HIV-infected children with invasive pneumococcal disease are more likely to have a prior diagnosis of AIDS, to have penicillin-resistant pneumococcal strains and to experience recurrence of invasive pneumococcal disease [9,12,21].

**F. Pneumococcal conjugate vaccine trials -potential for intervention**

The above illustrates the need for intervention beyond antimicrobial therapy in dealing with invasive pneumococcal disease. As early as 1945, Macleod et al showed that capsular polysaccharide used as vaccines could prevent pneumococcal pneumonia. The efficacy of polysaccharide vaccines is, however, limited among infants and young children because of their inability to mount an efficient T-cell-independent response to most polysaccharide antigens [23-26]. This problem has been overcome by the development of conjugate vaccines. The protein moiety in such vaccines allows it to be processed in a T-cell dependent manner, enhancing its immunogenicity in young children.

The successful use of this technology has been demonstrated with *H. influenzae* type b [Hib] conjugate vaccines. These vaccines are safe, immunogenic, and induce boostable memory responses in children under 2 years of age. Hib conjugate vaccines have also been shown to be clinically efficacious [5,26-30] and to reduce nasopharyngeal carriage of *H. influenzae* type b in vaccinated children [27,30-32]. The latter has important implications for invasive pneumococcal disease, since invasive pneumococcal disease usually occurs within 4 weeks of colonization of the nasopharynx by a new *S. pneumoniae* strain.

Similar conjugate technology has also been applied to the development of pneumococcal vaccines. Presently a number of Phase II studies of multivalent conjugated pneumococcal vaccines have been completed and/or are nearing completion in children; the vaccines are safe and immunogenic [33,34]. King et al showed the pneumococcal conjugate vaccine to be more immunogenic than the pneumococcal polysaccharide vaccine in both HIV-infected and uninfected children; however, the antibody response to both vaccine types was lower in HIV-infected children than in HIV-uninfected children [2]. Among HIV-infected adults the pneumococcal conjugate vaccine offers no advantage to the 23-valent *S. pneumoniae* polysaccharide vaccine. HIV-infected patients with CD4-cell counts greater than 500 cells/ml, showed a similar antibody response to HIV-uninfected adults.

Three large-scale Phase III trials have started using a 7-valent pneumococcal conjugate vaccine produced by Wyeth-Lederle Vaccines and Pediatrics [WLVP]. In Finland, Eskola et al are evaluating the efficacy of the vaccine to prevent otitis media. Investigators from the Northern California Kaiser Permanente Pediatric Vaccine Study Center are also testing the vaccine to decrease the incidence of invasive pneumococcal disease. A Phase III cluster-designed study of the 7-valent vaccine is also in progress in Navajo and Apache Native American children in the United States.

A new nonavalent pneumococcal conjugate vaccine for global use has been developed by WLVP. A Phase II trial conducted by Klugman et al, SAIMR, involving 500 infants given either the new vaccine or placebo is reaching completion at Zola Clinic, Soweto, South Africa. Preliminary results show the vaccine to be well-tolerated and immunogenic and to reduce the nasopharyngeal carriage of vaccine-associated serotypes. A Phase III trial is proposed to evaluate the efficacy of the same nonavalent pneumococcal conjugate vaccine in reducing pneumonia and invasive pneumococcal disease in children in the Soweto area. The potential efficacy of this vaccine in HIV-infected infants will also be assessed.

**G. Pneumococcal serotype\serogroup distribution and potential vaccine coverage**

Geographic and temporal variations in the distribution of *S. pneumoniae* serogroups\types necessitate the design of customized pneumococcal conjugate vaccines. In a study by Anderson et al it was estimated that the heptavalent vaccine would potentially cover 80% of serotypes responsible for invasive pneumococcal disease in the United States [33]. Sniadack et al, in a meta-analysis, determined that the optimal nonavalent pneumococcal conjugate vaccine for global use should include serotypes 1, 5, 6B, 7F, 9V, 14, 18C, 19F and 23F. In developed countries, the important serogroups (in descending order of frequency) are 14, 6, 19, 18, 9, 23, 7, 4 and 1; in developing countries 6, 14, 8, 5, 1, 19, 9, 23, 18, 15, 7 are important [35]. The temporal and geographic variation of serogroups\types demonstrate the potential pitfalls of pneumococcal conjugate vaccines which are designed independent of epidemiological data.

Between 1979-1986 the most prevalent serogroups among all patients in South Africa were 1, 6, 19, 14, 8, 3, 7, 4 and 18 [36]. More recent data among children at Chris Hani Baragwanath Hospital show the following serogroups to be of high prevalence: 6, 14, 1, 19, 23, 15, 19 and 3. The most important penicillin-resistant pneumococcal strains belong to serogroups 6, 23, 14 and 19; 73.3% of children with penicillin-resistant pneumococcal strains were HIV-infected [12].

Based on additional data from Friedland et al [15], a nonavalent vaccine which includes *S. pneumoniae* serogroups/types 1, 4, 5, 6B, 9V, 14, 18C, 19F and 23F would cover 90% of the serotypes responsible for invasive pneumococcal disease at Chris Hani Baragwanath Hospital pediatric department.

**H. Description of pneumonia surveillance study**

A prospective study examining the etiology of acute lower respiratory tract infections and the spectrum of invasive pneumococcal disease is currently underway at Chris Hani Baragwanath, Coronation, and Johannesburg Hospitals. During the first four months of the study, 351 children have been enrolled in the study; 79% of the children are from Soweto or Orange Farm, the catchment area for the efficacy study, and at least 80% of the children are up-to-date with their immunizations. HIV-seropositivity has been documented in 109 (31%) patients, 114 (32%) are HIV-seronegative and the remaining 128 (37%) are of unknown HIV status but are clinically negative, i.e., do not have signs/symptoms of HIV infection. The mean age of children admitted with pneumonia is 11 months; the mean age of the children did not differ by HIV status.

A virus, predominantly respiratory syncytial virus, has been isolated from 50% of the HIV-seronegative children and 23% of the HIV-infected children. Bacteria have played a larger role in pneumonia in HIV-infected children compared with children not infected with HIV (28% versus 8%). Pneumococci have been the most common bacteria isolated from the blood of children with severe pneumonia, accounting for 60% and 50% of the bacterial isolates from HIV-seronegative and HIV-seropositive children, respectively. *Haemophilus influenzae* type b has been the second most common bacterial isolate and has been significantly more common in children with HIV infection. In this study the case-fatality rate from pneumonia is presently 3% for HIV-uninfected children and 18% for HIV-infected children.

These preliminary data suggest an important role for *S. pneumoniae* as a cause of severe pneumonia in children in South Africa. Based on enrolment figures from the first 4 months, an estimated 1200-1400 children under 5 years of age will be recruited by the end of the study. These data should provide a more accurate measure of the incidence of acute lower respiratory tract infection which will be used to calculate sample size requirements for the Phase III pneumococcal conjugate vaccine study.

**II. Study objectives**

**A. The primary objectives of the study**  **are:**

1. To determine the protective efficacy of the nonavalent conjugate pneumococcal vaccine against invasive pneumococcal disease due to serotypes in the vaccine, in HIV-uninfected (HIV ELISA negative or HIV ELISA positive/HIV PCR negative) children who have received three doses of the vaccine per protocol (see Section VIII for definition). Invasive pneumococcal disease will be defined as isolation of *S. pneumoniae* from any normally sterile body fluid, including blood, cerebrospinal fluid, pleural fluid, ascitic fluid, and synovial fluid. Only the first episode of disease will be included in the analysis and the cases must occur ≥ 14 days after the 3rd dose of study vaccine.

2. To determine the clinical efficacy of the conjugate nonavalent pneumococcal vaccine in reducing radiologically-confirmed severe pneumonia (see definition Section VIII) in hospitalized HIV-uninfected children who have received three doses of the vaccine per protocol. To qualify for the primary objective, hospital admission must occur ≥ 14 days after the 3rd dose of study vaccine.

**B. The secondary objectives of the study**  **are:**

**HIV-uninfected children**

1. To evaluate the efficacy of the pneumococcal conjugate vaccine on the incidence of invasive pneumococcal disease due to all pneumococcal serotypes in HIV-uninfected children who have received three doses of study vaccine per protocol.

2. To evaluate the efficacy of two or more doses of the pneumococcal conjugate vaccine against vaccine serotype-specific invasive disease and against hospitalized pneumonia.

3 To determine the protective efficacy of the pneumococcal conjugate vaccine against vaccine serotype-specific invasive disease in an ‘intent-to-treat’ analysis, i.e., analysis including all randomized children receiving at least one dose of study vaccine.

4. To further assess the safety and immunogenicity of the conjugate pneumococcal vaccine.

5. To derive a population-based serological correlate of protection.

**HIV-infected children (HIV ELISA positive and confirmed positive by PCR)**

1. To evaluate the efficacy of pneumococcal conjugate vaccine on the first episode of invasive pneumococcal disease, as previously defined, caused by vaccine serotypes in HIV-infected children receiving three doses of study vaccine per protocol.

2. To evaluate the efficacy of pneumococcal conjugate vaccine on the incidence of invasive pneumococcal disease caused by all serotypes in HIV-infected children who have received three doses of study vaccine per protocol.

3. To evaluate the efficacy of pneumococcal conjugate vaccine on the incidence of pneumonia in hospitalized HIV-infected children who have received three doses of study vaccine per protocol.

4. To evaluate the efficacy of two or more doses of the pneumococcal conjugate vaccine on the incidence of vaccine serotype-specific invasive disease and pneumonia in HIV-infected children.

5. To assess the impact of pneumococcal vaccination on pneumonia-related and all cause mortality and time to death of hospitalized HIV-infected children.

**III. Study site**

The study will be conducted in South Africa in Soweto and in the informal settlement of Orange Farm, just outside of Soweto. Based on data from a recently completed population survey, it is estimated that children under 5 years of age account for 11.8% of the total population of 1,02 million people [13]. A national census conducted in 1996 should allow us to delineate the demographics of this community in even greater detail.

Although there is an emerging middle-class in the community, the majority of the population is still considered to be of lower socioeconomic status. Unemployment is estimated at 40% and there is a high incidence of teenage pregnancies. Risk factors that predispose to pneumonia include overcrowded living conditions, indoor and outdoor pollution, exposure to passive smoking, overcrowded crèche facilities and an increasing HIV infection rate.

There are 12 major clinics and 5 mobile clinics in Soweto and 4 clinics in Orange Farm providing free primary health care to all children; some of these clinics also provide routine immunization services. Patients who require admission are referred to Chris Hani Baragwanath Hospital, an academic hospital associated with the University of the Witwatersrand. The hospital has in excess of 2300 inpatient beds, 200 of which are used for general pediatric admissions. Approximately 24,000 babies are born in Soweto annually, two-thirds of which occur at Chris Hani Baragwanath Hospital and the remainder at one of six health centers equipped for obstetrics. A few children that require admission may attend the two other pediatric departments associated with the University, namely Coronation and Johannesburg hospitals, both of which are based in close proximity to Soweto and are easily accessible by public transport. Each of these hospitals has 50 general pediatric beds; all of the hospitals have X-ray and comprehensive laboratory facilities.

In South Africa immunization of all children is performed at Government health centers; the national immunization coverage in the first year of life is estimated at 74% [18]. In Soweto the immunization coverage is approximately 97% [Dr Kapongo- manuscript in preparation]. The recent Phase II study of pneumococcal conjugate vaccine at Zola Clinic had a 98% compliance rate over the first 3 doses of vaccine.

In the proposed study, vaccination with pneumococcal conjugate vaccine will be performed at all clinics providing routine immunization services in Soweto and Orange Farm [Appendix 3]. Vaccination will be performed by nurses trained in the administration of childhood immunization and who are specifically employed for this trial.

Surveillance for study patients admitted to Chris Hani Baragwanath Hospital will be done by study pediatricians and assistants in collaboration with the attending pediatricians at the hospitals.

**IV. Subject eligibility**

Infants who present to one of the health centers providing routine immunization services in Soweto and Orange Farm will be eligible for study participation based upon the following criteria. To be eligible for inclusion and continued participation in the study the child must fulfil all of the inclusion criteria and have none of the exclusion criteria

**A. Inclusion Criteria**

1. Infants who are 5-12 weeks of age.

2. Signed parental consent following a detailed explanation of participation in the study.

**B. Exclusion Criteria**

1. Any child who has already received his/her first DTP immunization.

2. A child with a progressive neurological disorder or a history of uncontrolled epilepsy/ infantile spasms.

3. A child unlikely to receive 3 doses of the vaccine in the study area.

**C. Conditions which might require delaying administration of vaccination**

A child with an intercurrent illness that requires referral/admission to hospital.

**D. Contraindications for subsequent doses of the study vaccine**

1. An immediate anaphylactic or any other serious reaction to a prior dose of study vaccine.

2. Child is withdrawn from the study at the request of the parent/guardian, or as a recommendation from the Safety Monitoring Committee.

3. Development of a progressive neurological or seizure disorder.

4. Contraindications specified in the manufacturers’ package insert for DTP/Hib vaccine [TETRAMUNE ®, Appendix 5]

**V. Trial design**

**A. Enrolment**

All children presenting to immunization clinics in Soweto or Orange Farm [Appendix 3] for routine immunization at approximately 6 weeks of age will be evaluated for study participation. Children will qualify for enrolment by meeting all inclusion criteria and none of the exclusion criteria, as determined by obtaining a medical history and performing a physical examination prior to immunization. The parent/guardian must also give consent for the child to participate in the study. Staff hired specifically for the study will do recruitment, immunization (study and routine vaccines), and follow-up.

The annual birth cohort in Soweto is approximately 24,000. It is anticipated that the entire birth cohort over a two-year period will be recruited.

**B**. **Randomization**

Randomization will occur immediately after consent for study participation has been obtained from a parent/guardian and study eligibility confirmed. Subjects will be randomized in equal numbers to receive either the pneumococcal conjugate vaccine or placebo; the unique subject identification numbers will be assigned in sequence. Due to some mobility within Soweto, children may not always receive their routine immunizations at the same clinic. To facilitate this movement, children will be randomized at the time of their first immunization to one of 10 vaccination codes, designated 0-9. The vaccination code and subject ID number will be recorded on the child’s Road to Health card, on the clinic medical records, and on the clinic immunization register. Immunization sites will be provided with vials for each of the 10 vaccination codes, thereby allowing children to receive their study immunizations at any of the study sites. Randomization schemes for each recruitment site will be generated prior to the start of the study by a statistician from WLVP who is otherwise uninvolved with the study.

**C. Blinding**

This study is double-blind. Pneumococcal conjugate vaccine and placebo will be supplied in lyophilized form in precoded vials by WLVP. TETRAMUNE® will be supplied by WLVP in commercial vials and hepatitis B (Hep B) and oral polio vaccines (OPV) will be supplied according to usual practice in South Africa. The identity of the ten vaccination codes (placebo versus vaccine) will be maintained in confidence from the investigators, study nurses and other study staff, from the clinical monitors, and from the child’s parents/guardians. The Road to Health cards, although usually brought to the hospital with the child, are not kept as part of the hospital bed letters; therefore, the study pediatricians will be blinded to the code of the vaccine received by hospitalized study participants. In the event that safety issues warrant the unblinding of an individual subject or the entire study, the identity of the vaccination codes will be kept by a member of the Safety Monitoring Committee (Dr. S.S. Abdool-Karim) and by an individual at WLVP not involved with the vaccine trial.

**D**. **Treatment period**

Each study subject will receive three injections of either pneumococcal conjugate vaccine or placebo, administered concurrently with TETRAMUNE®, OPV, and Hep B. The injections will be administered at approximately 6, 10, and 14 weeks of age, with a minimum interval of 3 weeks between each dose and all three doses must be completed by 9 months of age. Children not returning within 1 week of the appointed time for the second or third vaccinations will either be telephoned or visited at home.

**E. Control group**

In an effort to offer a potential benefit to those children randomized to receive placebo, Hib conjugate vaccine, included in TETRAMUNE®, will be given to all children in the study. Hib conjugate vaccine is not currently provided by the Expanded Programme on Immunization in South Africa. In addition, all of the children in the study who are admitted to the hospital with suspected infectious disease will receive an extensive diagnostic work-up to determine the etiology of their disease. The placebo group will serve as a control for evaluating the protective efficacy and safety of the nonavalent pneumococcal conjugate vaccine.

**F. Follow-up**

**1. Vaccine Safety**

Following each vaccination, the child will be monitored at the clinic for approximately 15 minutes for any immediate local and/or systemic reactions. The parent/guardian will also be asked to observe for untoward effects which might occur within one week of vaccination. If the parent/guardian has concern, they will be asked to report these reactions to the study nurse as soon as possible. Any child with a clinical event of concern to the parents will be evaluated by the study nurse or other medical personnel. In the event that the child is hospitalized, the parent will be asked to contact the investigator immediately. Study pediatricians will also be conducting daily surveillance for hospitalization of study participants. Participation in the vaccine study will be noted on the child’s Road to Health card, which is presented at hospital visits.

All efforts will be made to obtain follow-up information on deaths which occur outside of the hospital through death notifications, cemetary records, etc..

The frequency of adverse events following vaccination will be assessed for both the placebo and treatment groups.

**2. Vaccine Immunogenicity**

To confirm the immunogenicity of the vaccine, a subset of 200 children will be randomly-selected from the pool of all children who have received 3 doses of the vaccine or placebo at any one of the study clinics. For logistical reasons, only those children receiving all 3 doses of the vaccine at the same clinic will be eligible for the immunogenicity study. At the 14 week visit, the parent/guardian will be asked to consent to have blood taken from their child at 18 weeks, 4 weeks after the third immunization. Serum antibody responses to the pneumococcal vaccine will be measured by standardized ELISA methods. Anonymous and confidential HIV testing will also be performed to obtain an estimate of the HIV seroprevalence of children in the study. The randomization scheme for selection of children to participate in the immunogenicity study will be developed by WLVP prior to initiation of the trial. The data from the immunogenicity study will be kept confidential from the study investigators until the end of the trial; however, the information will be provided to the Safety Monitoring Committee.

The immunogenicity study will be repeated when each additional lot of pneumococcal conjugate vaccine is introduced into the study.

**3. Vaccine Efficacy**

The statistical methods for calculating the efficacy of the nonavalent pneumococcal conjugate vaccine are described in Section XII. Vaccine efficacy will be defined as the number of cases of culture-confirmed invasive pneumococcal disease or pneumonia in the HIV-negative group receiving the pneumococcal conjugate vaccine compared to the number of cases occurring in the placebo group. The study pediatricians will conduct daily surveillance for cases of severe pneumonia, as defined in Section VIII, at Chris Hani Baragwanath Hospital. In addition, a list of all cases of culture-confirmed pneumococcal disease will be obtained daily from the hospital microbiology laboratories. HIV testing will be performed in an anonymous manner for all children with study outcomes who are not tested for HIV during hospitalization. Those outcome cases with no HIV test results will be excluded from the efficacy analyses.

**G. Termination of follow-up for outcome surveillance**

Follow-up for any individual child will end when:

1. The study participant dies.

2. The Safety Monitoring Committee recommends early termination of the trial due to questions of vaccine safety or lack of efficacy.

3. The follow-up of study participants has been completed and the study is terminated.

4. If the child is lost to follow-up (moved out of the study area) and efforts to determine outcome status have been unsuccessful.

**VI. Conduct of the study**

**A. Enrolment**

1. Children approximately 6 weeks of age, operationally defined as 5-12 weeks of age, will be recruited at clinics performing routine immunizations in Soweto and Orange Farm. A list of the clinics is found in Appendix 3.

2. Prior to study enrolment, the child must undergo a physical examination and have a medical history obtained to ensure that he/she meets all inclusion criteria and none of the exclusion criteria.

3. The parent/guardian accompanying the child to the clinic will be informed of the study and, if they agree to have their child participate, will be asked to read and sign the informed consent. The informed consent will be reviewed and approved by the University of the Witwatersrand Committee for Research on Human Subjects and WLVP and will be available in English, Sesotho, Zulu, and Xhosa (see Appendix 6).

4. Children meeting the enrolment criteria will be randomized at each study site by assignment of the next sequential study identification number and corresponding vaccination code from a preprinted randomization list. Stickers indicating study participation, the study identification number, and the child’s vaccination code will be put on the child’s clinic medical records and on the Road to Health card. A separate Immunization Register containing the child’s and mothers name, address, child’s birthdate, study identification number, vaccination code, and dates of individual vaccine doses will be kept at each clinic.

**B.** **Administration of initial and subsequent vaccinations**

1. A medical assessment will be conducted before administration of each dose of test vaccine to ensure that the child continues to meet all of the inclusion criteria and none of the exclusion criteria.

2. The study nurse will determine which study vaccine to give from the Road to Health card. If the card is not brought to the clinic, the vaccination code will be determined from the clinic immunization register. In the event that the child presents to a different clinic and the Road to Health card is not available, the nurse will telephone either the clinic which initially randomized the child or will call the data management center who will determine the child’s vaccination code from the study computer database.

3. The preferred schedule is for subjects to receive their vaccines concurrently according to routinely scheduled vaccine timetables (see below), i.e., at 6, 10, and 14 weeks of age, with a minimum interval of 3 weeks between immunizations. The 2nd and/or 3rd doses of pneumococcal conjugate vaccine/placebo, however, can be given nonconcurrently from the other vaccines. All three doses of the conjugate vaccine/placebo must be completed by 9 months of age. The above constitutes “per protocol” vaccination schedule.

|  | **BCG** | **Tetramune** | **Hep B** | **OPV** | **Pneumo/**  **placebo** | **Measles** |
| --- | --- | --- | --- | --- | --- | --- |
| **Birth** | ***** |  |  | ***** |  |  |
| **6 weeks** |  | ***** | ***** | ***** | ***** |  |
| **10 weeks** |  | ***** | ***** | ***** | ***** |  |
| **14 weeks** |  | ***** | ***** | ***** | ***** |  |
| **9 mos** |  |  |  |  |  | ***** |
| **18 mos** |  | ***** |  | ***** |  |  |

The 0.5 ml of TETRAMUNE and 0.5 ml of Hep B vaccines will be administered intramuscularly using separate syringes with a 23 or 25-gauge needle in the anterio-lateral aspect of the right upper and right lower thigh respectively. The 0.5 ml of the trial vaccine will be administered in the anterio-lateral aspect of the left thigh. OPV will be orally administered. Standard immunization practices will be ensured. Epinephrine 1:1000 (1 mg/ml) and resuscitative equipment will be available in the event of an anaphylactic reaction. Vaccination Report Forms will be completed by the study nurse after each immunization.

4. The individual immunization clinics in Soweto are experienced in following children who have not returned for their scheduled routine vaccinations. Immunization registers will be used for follow-up of subsequent vaccinations. Each day the study nurse will review the register, record those patients who have been immunized on that day, and identify those patients who failed to attend. The study nurse will also be provided with a weekly list of all children enrolled at their site who were immunized the previous week at another study site. If telephone numbers are available, the study nurse will telephone parents of children who have failed to attend and remind them of the child’s clinic appointment. If no telephone number is available, the study nurse will make a home visit to the family. If a subject is withdrawn from the study, the circumstances will be documented in the Vaccination Report form.

5. In the rare cases of errors in vaccinating a child with vaccine of the wrong code (deviation from the randomization schedule), the subsequent doses should be the vaccine of the same code as the first dose. These children will not be included in the primary efficacy analysis. They will be included in the intent-to-treat analysis in the group based on the vaccine they receive as the first dose.

**C. Immunogenicity study**

A list of children to participate in the immunogenicity study will be generated by WLVP and will include subjects who have received either the conjugate vaccine or placeboat any one of the study sites. One nurse will be specifically responsible for obtaining blood specimens from the appropriate children; therefore, for logistical reasons, only children who have received all 3 doses of the vaccine/placebo at the same clinic will be eligible for the immunogenicity study. As there is no scheduled clinic visit at 18 weeks, the informed consent will be given to the parents at the 14 week visit. To ensure that blood is taken from appropriate numbers of children given either placebo or conjugate vaccine, the study identification numbers of children initially randomized into the immunization study who, for any reason, are not bled, will be sent to WLVP. A list of alternate children will then be generated.

Approximately 2 ml of blood will be taken by finger stick at 18 weeks, 4 weeks after the third immunization. The blood will be collected in a red tube and then the sera will be removed after clotting and centrifugation and stored at -70^o^C.

**D. Assessment of vaccine safety**

1. All children will be observed for 15 minutes following vaccine administration for any immediate local and/or systemic reactions at the clinic by qualified study personnel. Parents will also be advised to return to the clinic immediately should the child develop reactions of concern to the parent. Follow-up will occur on all allergic reactions, severe local reactions within 72 hours of vaccination, as well as on all seizures occurring within 4 weeks of vaccination.

2. The study pediatricians will conduct daily surveillance for hospitalization of study children (see Section VIII) at Chris Hani Baragwanath Hospital. All deaths, life-threatening events, and unexpected events related to the vaccine will also be investigated. Efforts will also be made to follow-up all deaths occurring outside of the hospital through death notifications and cemetery records. Prior to implementation of the study, meetings will be held with the pediatricians of the hospitals to inform them of the nature of the study and the need to notify study investigators should they see study participants. Reminder notices will be placed in the appropriate wards and the children’s Road to Health cards will also be clearly labelled so as to indicate their participation in the study. The majority of mothers bring the Road to Health card when the child is hospitalized.

3. The Safety Monitoring Committee (see Section XIV) and WLVP will review safety data quarterly during the trial. All severe, life-threatening reactions or the deaths of study participants, regardless of the etiology, and unexpected events felt by the study physicians to be vaccine-related will be reported monthly to the members of the Committee as well as to WLVP. Quarterly reporting to WLVP will include all of the above reactions as well as seizures or changes in neurological status within one month of vaccination and severe local or systemic reactions or allergic reactions within 72 hours of vaccination. A report of the incident cases of pneumococcal disease will be provided to the Safety Committee quarterly.

**E. Assessment of vaccine efficacy**

**1. Identification of study participants admitted to hospital** **with possible infectious disease etiology**

All pediatric patients under 2½ years of age, adjusted for the duration of time the study has been in progress, will be screened upon arrival in the admission ward to determine whether the patient has been enrolled in the study. The admission ward will be staffed on a 24-hour basis by qualified nursing assistants, who will interview the escort of the child to determine if the child is resident in the study area and has been recruited in the study. This information will be verified by reviewing the child’s Road to Health card. If the card is not immediately available, the escort will be requested to bring the card at his/her next visit. Further confirmation of study participation will be done through the central database. The name of the child, the date of birth, address, and mother’s name will be obtained from escorts of all admitted patients within the study age range. This information will be sent to the data management center for cross-checking with the central database of all study participants.

To ensure that all hospitalized study participants are assessed by study physicians, the register in each admitting ward will be reviewed daily for any children within the appropriate age range. Escorts of these children will be interviewed as described above.

All patients will have a Case Information Form completed after being assessed by a study physician; forms from patients identified as study participants will be sent to the data management center. All efforts will be made, however, to blind the study physicians to the vaccination code of the cases. Study physicians will not see the Road to Health cards. Case Information Forms from non-study patients will be stored separately and will be cross-checked with the central database for a period of two weeks. This is to ensure that children whose forms may have been delayed in reaching the data management center or not have been immediately entered into the database are not misclassified as non-study participants.

Further surveillance by study physicians will include follow-up of all patients in the pediatric wards from whom *S. pneumoniae* has been isolated from a normally sterile body site, as reported by the hospital microbiology laboratory. Case Information Forms, etc will be completed and verified as described above.

**2. Investigation of hospitalized study-patients**

**a. Routine investigations**

Investigations will be determined by the clinical presentation of the child; however, the following tests are routine:

* full blood count

* blood culture

* C- Reactive-protein

* Chest X-ray if pneumonia/septicaemia is suspected

* Lumber puncture for cerebro-spinal fluid analysis if meningitis is suspected

* Other test/s as recommended by the attending pediatrician

**b. Additional tests that are specific to the study**

A total of 2-3 ml of serum will be collected in a red tube for pneumococcal antibody titer testing; antibody titers will be performed concomitantly at SAIMR and/or at WLVP using standardized ELISA tests.

A second blood specimen of 2 ml will be taken in an EDTA (purple) top tube for HIV testing. All clotted blood will be stored refrigerated and clarified by centrifugation within 24 hours after collection at the central SAIMR laboratory. Approximately 0.6 ml of clarified serum will be transferred into 1.0 ml tubes and labeled with the appropriate patient identity. All sera will be stored in a -70^o^C freezer. HIV ELISA testing will be performed on serum from those children who were not tested during hospitalization. The serum will be renumbered and linked to non-identifying features eg, number of doses received, age at time of disease, nature of disease, etc. To confirm HIV positive status, HIV PCR will be performed on all serum found to be ELISA positive in the hospital as well as during the testing at SAIMR. Children with HIV ELISA positive/PCR negative serum will be considered to be HIV-uninfected.

**c. Microbiology methods**

Isolates of *S. pneumoniae* will be identified by standard microbiological techniques at the microbiology laboratory of the hospital to which the patient has been admitted, i.e., the microbiology laboratories at Chris Hani Baragwanath Hospital. Serotyping/serogrouping of pneumococci will be performed using both the Quelling and latex reactions at the MRC/SAIMR/Wits/Pneumococcal Diseases Research Unit. Antibiotic susceptibility testing will be performed using the disc diffusion method. Minimum Inhibitory Concentrations (MICs) will be determined using the microdilution method for those isolates that show resistance by the disc diffusion method.

**VII. Vaccines and vaccine management**

**A. Nonavalent pneumococcal conjugate vaccine**

The nonavalent conjugate vaccine is manufactured by WLVP. Each 0.5 ml. dose contains 2 μg each of polysaccharide belonging to serotypes 1, 4,5, 9V, 14, 19F and 23F; 2μg of oligosaccharide of serotype 18C; and 4 μg of 6B polysaccharide. All the polysaccharides are independently coupled to a total of approximately 20 μg of CRM 197 (a non-toxic mutant of diphtheria toxoid) that acts as a protein carrier.

**B. ‘Control’ vaccine**

Patients in the control group will receive a placebo injection that is identical in appearance to the nonavalent pneumococcal conjugate vaccine. The placebo will be prepared by WLVP and will contain sucrose, the stabilizer for the pneumococcal conjugate vaccine.

**C. Other vaccines administered at 6, 10 and 14 weeks**

All children will receive TETRAMUNE®, manufactured by WLVP, and Hep B (Hepacine, Cheil Sugar Organization, Korea) and OPV (Polioral trivalent, Biocine, Italy), which are the routine vaccines given to children in South Africa.

**D. Management of study vaccines**

Study vaccines will be shipped from the manufacturer to SAIMR in Johannesburg, South Africa. The vaccine will be locked in a secure storage facility and temperature control of this facility will be ensured. Vaccines will be brought by courier to the health centers involved in vaccination, where storage, safe-keeping and dispensing of the vaccine will be the responsibility of the study nurse. Daily recording of refrigerator temperature will be conducted and logs of the vaccine doses given each day will also be kept. The principal investigator and/or one of his appointees as well as WLVP monitors will oversee the management of the study vaccines. Comprehensive accounting will allow tracking of all vaccine doses from initial receipt to dispensing at the facilities.

**VIII. Case definition**

**A. Determination of efficacy against vaccine serotype invasive pneumococcal disease in HIV-uninfected children**

To be included in the first primary efficacy calculations the case must meet the following criteria:

* Have *S. pneumoniae* of a vaccine serotype isolated from a sterile body fluid, defined as blood, cerebrospinal fluid, pleural fluid, ascitic fluid, and synovial fluid, 14 days or more after the third dose of study vaccine.

* Have been vaccinated according to the protocol (see Section VI).

* Not have acquired or congenital immunodeficiency syndromes (HIV negative status determined by PCR for those with a positive HIV ELISA).

* Be the first episode of invasive pneumococcal disease of any serotype.

**B. Determination of efficacy against pneumonia in HIV-uninfected children**

To be included in the second primary efficacy calculations the case must meet the following criteria:

* Admission to hospital for pneumonia 14 days or more after the third dose of study vaccine.

* Have been vaccinated according to the protocol (see Section VI).

* Not have acquired or congenital immunodeficiency syndromes (HIV negative status determined by PCR for those with a positive HIV ELISA).

Patients enrolled in the study who require hospitalization will be categorized according to the following system, based on their clinical presentation.

**C. Clinical definition of cases**

**1. Hospitalized pneumonia**

This definition relates to efficacy against pneumonia as it will appear in the primary analysis. This is a radiological definition in recognition of the subjectivity of the clinical signs of pediatric pneumonia. The usual clinical signs, tachypnea, chest wall indrawing, fever, crepitations, and rhonchi can all be present in acute infections other than pneumonia. More specific auscultatory signs, such as bronchial breathing are not very sensitive and are unlikely to be found in the absence of striking radiological signs. Furthermore, children with definite radiological pneumonia without any of the usual clinical signs are found frequently and are generally regarded as having pneumonia. Radiological pneumonia will be defined as one or more of the following:

* definite lobar consolidation

* definite diffuse or non-lobar consolidation

* pleural fluid

All readings will be made by a pediatrician with experience in reading chest radiographs and no clinical information about the case. Only radiographs taken during the admission in question will be viewed, but all radiographs may be viewed. All cases positive by the above criteria will be confirmed by a second reader. If there is not agreement that a case is positive, it will be regarded as negative. When a child is discharged or dies, the radiographs will be set aside to be read at the next reading session. Reading sessions will take place every 2 weeks.

**2. Graded ARI definitions**

These definitions are intended for use in the secondary analyses, to determine the contribution of pneumococci of vaccine serotypes to various severities of ARI, and the efficacy of the vaccine for the prevention of various ARI syndromes.

**a. Acute lower respiratory infection - non severe**

Cough of less than 14 days duration associated with fast breathing ( >50/min in children less than one year of age and >40/min in children older than one year). No lower chest wall indrawing, respiratory distress, inability to drink, cyanosis, or desaturation (SaO_2_ < 90%). The child is not in need of admission for this problem alone.

**b. Severe pneumonia**

Cough of less than 14 days duration with any one of the following: indrawing of the lower chest wall (subcostal retractions) in well-nourished children and/or intercostal retractions in malnourished children, respiratory distress, inability to drink, cyanosis or desaturation (SaO_2_ <90%), *and* with a chest radiograph positive for pneumonia by the above criteria.

**c. Severe acute lower respiratory infection**

Any child whose primary symptom is cough of less than 14 days duration and who is judged on clinical ground to require admission to hospital. Acute laryngotracheobronchitis (croup) and other severe upper respiratory tract infections are excluded from this category.

**d. Very severe pneumonia**

Any child with confirmed radiological pneumonia who also has hypoxemia (SaO_2_ < 90%).

**3. Pneumococcal pneumonia**

A diagnosis of pneumococcal pneumonia will be made if the child fulfils the above clinical and/or radiological criteria and if *S. pneumoniae* is isolated from blood or pleural fluid.

**4.**  **Pneumococcal meningitis**

Pneumococcal meningitis will be diagnosed if *S. pneumoniae* is isolated from CSF and/or if, in addition to the following clinical criteria, pneumococci are isolated from blood or the latex agglutination test on CSF is positive for pneumococci.

* CSF neutrophil count of >5 cells\ ml, and

* total CSF protein of >0.5 g\l, and

* CSF glucose <50% of blood glucose level

**5. Other invasive pneumococcal disease**

These will include the following diagnoses based on isolation of *S. pneumoniae* from blood and/or relevant normally sterile body fluids:

**a. Pneumococcal peritonitis**

The presence of abdominal tenderness and guarding in a child with purulent ascitic fluid.

**b. Pneumococcal septic arthritis**

The presence of clinical signs of joint inflammation and purulent synovial fluid.

**c.** **Pneumococcal cellulitis**

Inflammation of subcutaneous tissue.

**d.** **Pneumococcal septic shock without a focus**

The presence of fever, tachycardia coupled with abnormal responsiveness, and/or hypotension, and/or dehydration, and/or metabolic acidosis (pH <7.25)

.

**IX. Sample size and plan for analyses**

The study is designed primarily to evaluate the impact of the nonavalent pneumococcal conjugate vaccine in HIV-uninfected children. The cases in HIV-infected children will not be included in any primary analyses of vaccine efficacy as their outcome variables, disease incidence rates, expected vaccine efficacy, and expected follow-up time may all be different from that in the general population. The impact of the vaccine in this special population will be evaluated separately as a secondary objective. All case ascertainment will include HIV testing, in an anonymous and confidential manner. The HIV status will only be linked to clinical outcomes at the time of analysis. It will not be linked to any other health record of the child. Outcome cases for which HIV results are not available will be excluded from analyses.

**A. Primary outcome variables**

1. Cases (see Section VIII for case definition) of vaccine-serotype pneumococcal invasive disease in children who have completed the three-dose series per protocol.

2. Cases (see Section VIII for case definition) of radiologically-confirmed hospitalized pneumonia in children who have completed the three-dose series per protocol.

Outcomes will qualify for the primary analysis of they occur at least 14 days after the third dose of vaccine/placebo. Outcome ‘1' will be the basis for the vaccine efficacy estimate. Since the prevalence of radiographically-confirmed severe pneumonia may be several-fold higher than that of culture-confirmed pneumococcal invasive disease (to be estimated from the ongoing pneumonia surveillance study) and the mortality rate from severe pneumonia is probably high (to be estimated from the ongoing pneumonia surveillance study), outcome ‘2' will be used to assess the effectiveness of the vaccine in reducing the overall pneumonia disease burden and its impact on public health as well as to estimate the proportion of severe pneumonia caused by pneumococci of vaccine serotypes.

**B. Statistical analyses for the primary outcome variables**

Efficacy of the vaccine will be estimated by comparing the incidence rates of vaccine-serotype invasive pneumococcal disease (Primary Outcome ‘1') between the recipients of three doses of the vaccine and placebo. Efficacy will be estimated as (1-RR) x 100%, where RR is the ratio of the incidence rate of the treated group versus that in the control group. With individualized randomization of a large cohort at a 1:1 allocation ratio, the follow-up time in the treated and control groups should be equal. The rate ratio can, therefore, be estimated as the ratio of the number of cases in the treated group versus that in the control group.

An exact two-sided 95% confidence interval for vaccine efficacy will be estimated, based on the binomial distribution for the number of cases among vaccinees, given the total number of cases and the ratio of follow-up time in placebo recipients to follow-up time in vaccine recipients. The statistical power for this analysis depends on the true efficacy and the number of accrued cases. For power calculations, we assume the true efficacy against vaccine-serotype invasive disease is 70% and that the ratio of follow-up times is 1. Then 28 cases are required for an 80% probability that the lower limit of the 95% confidence interval for efficacy will be greater than 0 (i.e., to reject the hypothesis of no efficacy); for an 80% probability that the lower limit will be greater than 20% 43 cases are required. We plan to enrol study infants until 28 HIV-uninfected vaccine-serotype cases have been accrued for the primary analysis and then continue surveillance until a total of at least 43 cases have been accrued. The following cases splits, out of the 28 cases, can lead to rejection of the null hypothesis.

| Control Cases | Treated  Cases | Estimate of vaccine  efficacy (%) | Lower 95% Confidence  Limit of VE (%) |
| --- | --- | --- | --- |
| 28 | 0 | 100 | 85.9 |
| 27 | 1 | 96.3 | 77.5 |
| 26 | 2 | 92.3 | 69.2 |
| 25 | 3 | 88.0 | 60.6 |
| 24 | 4 | 83.3 | 51.4 |
| 23 | 5 | 78.3 | 41.5 |
| 22 | 6 | 72.7 | 30.6 |
| 21 | 7 | 66.7 | 18.6 |
| 20 | 8 | 60.0 | 5.1 |

The current estimate of the incidence of invasive pneumococcal disease in children under the age of 2 years in Soweto is 160/100,000 child years [12]. Assuming that 70% of the disease cases are due to the vaccine serotypes, the vaccine-serotype disease rate is 112 cases per 100,000 child years. Based on this estimate of disease rate and the assumption that the true vaccine efficacy is 70%, approximately 19,231 child years follow-up per treatment group will be needed to accrue 28 cases of vaccine-serotype disease in HIV-uninfected children. The annual birth cohort in Soweto is approximately 24,000 infants, 4% (960) of whom would be expected to be HIV-infected. Allowing for loss to follow-up, the entire birth cohort over a two year period will be recruited to reach the targeted enrolment goal. The study is expected to accrue 28 cases of vaccine-serotype disease in HIV-uninfected children in approximately 32 months. Approximately 29,533 child years of follow-up per treatment group, expected in approximately 39 months, will be needed to accrue 43 cases. A more accurate estimate of the disease rate is expected to be obtained prior to the start of the study from the prospective cohort study currently underway in Soweto.

The rate of hospitalized pneumonia (Primary Outcome ‘2') will be compared between the treated and the control group using a Poisson regression, with age category and calendar month of the third dose (season category) as possible stratification variables. An estimate of the rate reduction and its confidence interval will be obtained from the Poisson regression. Multiple events of an individual will be included in the rate estimates. The null hypothesis is that there is no rate reduction and will be tested based on the Poisson regression with a two-tailed α = 0.05. An analysis to compare the number of children with at least one event between the two groups will also be performed.

The statistical power for the analysis of pneumonia (Primary Outcome ‘2') depends on the incidence rate in the control population and the reduction of the rate in the vaccinated population. Once a reliable estimate of the disease rate becomes available from the prospective cohort study, detailed power calculations for this outcome variable will be provided. However, since the disease rate is expected to be substantially higher than the rate for vaccine-serotype invasive disease, it is expected that by the time 28 cases of serotype-specific invasive disease are accrued, the study cohort will be enough to provide an excellent statistical power to detect a clinically significant reduction in the rate of severe pneumonia.

**C. Secondary efficacy outcome variables and statistical analyses**

The following efficacy outcome variables will also be evaluated for HIV-seronegative children. The analyses will include the graded ARI case definitions listed in VIII C.2. The primary outcomes (see above) and secondary outcomes 1 and 2 below will also be evaluated for children infected with HIV (see E below) as secondary analyses. The power for these analyses will depend on the number of outcome cases meeting the definition for inclusion in the particular analysis.

1. All invasive pneumococcal disease, including nonvaccine serotypes, in children who complete three doses of vaccine. This analysis will be performed using the methods and case definitions described above for the primary outcomes and include all first episodes of pneumococcal disease, regardless of serotype.

2. Vaccine-serotype invasive pneumococcal disease in children who complete at least two doses of vaccine, the first episode of disease occurring at least 14 days after the 2nd dose of vaccine. Exact binomial tests, including children who meet the case definitions described above and who have received either 2 or 3 doses of the vaccine, will be used as for the primary outcome analyses.

3. Vaccine-serotype invasive pneumococcal disease in all children who have received at least one dose of vaccine in the study regardless of immunization status (intent-to-treat analysis for vaccine-serotype pneumococcal disease). The intent-to-treat analysis will include the first episode of vaccine-serotype pneumococcal disease in children who were initially randomized into the study and received at least one dose of study vaccine and regardless of the number of vaccine doses they eventually received or whether they voluntarily withdrew from the study and were lost to follow-up. The null hypothesis of no difference between the two groups in all randomized chldren will be tested.

4. Vaccine immunogenicity will be assessed in a random sample of 400 children over the study period, or 200 children per lot of study vaccine introduced into the trial. The geometric mean titers of the antipneumococcal antibodies and 95% confidence interval of the vaccinated children will be determined and the distribution will be examined using reverse cumulative plots. The different response distributions of vaccine lots will be compared.

5. Vaccine safety will be assessed by adverse reaction reports of all study children, including all hospitalizations. Report rates of the vaccinated and control group will be compared using Fisher’s Exact test.

6. The relationship between vaccine efficacy and antibody response will be evaluated based on the distribution of antipneumococcal antibodies in vaccinated and control children.

**D. Other analyses**

Additional data analyses will include the following:

1. The impact of vaccination on the proportion of cases of invasive pneumococcal disease that are penicillin-resistant. The proportion in each vaccine group will be compared by Chi-square or Fisher’s Exact test.

2. The efficacy of the vaccine against invasive pneumococcal disease due to individual serotypes will be evaluated using exact binomial tests as described above if there are at least 6 cases of disease caused by an individual serotype.

3. The impact of the vaccine in the total pneumonia disease burden will be assessed by comparing the incidence of pneumonia requiring admission in vaccinees and controls using an exact binomial test.

**E. HIV-positive population**

It is estimated that 4% of the annual birth cohort (approximately 900 children) in Soweto will be HIV-seropositive. The effect of the vaccine on the following outcome variables in children enrolled in the study who are confirmed to be HIV-infected will be evaluated.

1. Vaccine-serotype invasive disease. The analysis will include the comparison of disease rates and time to first episode of disease (from 14 days after the third dose of vaccine) between the vaccinated and control children. A sensitivity analysis will be performed to examine the robustness of the vaccine efficacy estimate with respect to the assumption of equal follow-up time in the two groups.

2. Radiologically-confirmed hospitalized pneumonia. The analysis will include the comparison of event rate and time to first event between the two groups. An analysis to compare the number of children with at least one event will also be performed.

3. All invasive pneumococcal disease, including nonvaccine serotypes. The analysis will be done as described in Section IX.C.1 above.

4. Pneumonia-related mortality. Mortality resulting from pneumonia will be analyzed using the life table method and the survival curves of the vaccinated and the control group will be compared using the log rank test.

5. All cause mortality. The all-cause mortality will be analyzed using the life table method and the survival curves of the vaccinated and the control group will be compared using the log rank test.

**F. Estimated study mortality**

Using statistics from Chris Hani Baragwanath Hospital, 50% (450) of the HIV-infected children enrolled in the study will die within the first year of life. The infant mortality rate for children between 6 weeks and 2 years of age in Soweto is approximately 10/1000, suggesting that an additional 220 children will also die during one year of follow-up. In total, an estimated 670 children in the study cohort will die per annum.

**G. Interim analysis**

No formal interim analysis of the efficacy data is planned and no early termination of the study due to early evidence of efficacy is planned. Once 28 outcome cases have been accrued, enrolment into the study will stop. The International Advisory Committee (Section XIII.A) will then meet to discuss whether/how long surveillance for outcome cases should continue. Follow-up will likely occur until 43 cases have been accrued. Quarterly reports of safety data, including adverse events and deaths, and reports of cases of pneumococcal disease and pneumonia will be reviewed by the Safety Monitoring Committee (SMC). Conditional power calculations or the probability of ever showing significant efficacy given the observed number of outcome cases in each vaccine group and an assumed level of vaccine efficacy, may be provided if requested by the SMC. The study could be terminated early only if one of the following is concluded by the SMC:

(1) significant safety concern

(2) increased pneumococcal disease risk in the vaccinated group, or

(3) extreme low probability of demonstrating efficacy even if the study is carried through to the end

**X. Adverse reactions**

The results of the Phase II trial assessing the safety and immunogenicity of the nonavalent pneumococcal conjugate vaccine, which is presently being concluded at Zola clinic, will be available soon. Preliminary data from 500 children suggest the vaccine to be well-tolerated. No serious adverse reactions were recorded. During the proposed efficacy study all children will be monitored for 15 minutes after vaccination for any signs or symptoms of adverse reactions. Resuscitation facilities will be available at all the health centers. All signs or symptoms will be recorded on an Adverse Reaction Form as well as on the Road to Health card.

Parents will also be advised to watch for signs and symptoms of local reactions, including erythema, swelling, induration and/or tenderness at the site of vaccination and for systemic reactions, such as difficulty with breathing, drowsiness, vomiting and fever lasting for greater than 24 hours. Parents will be instructed to bring their children to the clinic/hospital immediately for medical assessment and/or management for any vaccine event of concern.

Parents/guardians will be asked to request doctors/nurses not directly involved with the study to note any event requiring medical attendance on their Road to Health card, including the clinical diagnosis and treatment given. The Road to Health card, which is usually safeguarded by the parent/guardian, will be reviewed at each vaccination visit. The card will also be reviewed by the admitting physician should the child require hospitalization.

Any patient experiencing a serious clinical adverse reaction will have a detailed history, physical examination and appropriate laboratory procedures done by a study pediatrician. All deaths, life-threatening events, and all unexpected vaccine-related events will also be investigated. These events will be reported to the Safety Monitoring Committee and WLVP monthly.

**XI. Data management and data quality control**

Each Vaccination Report form will be reviewed and initialed by the study nurse. Once reviewed by the clinical monitors, the forms will be brought to SAIMR for entry into a SAS computer program. Data from the Vaccination Report forms, the Adverse Reactions forms, the Clinical Outcome forms, and Laboratory forms will be entered onto desk-top computers and verified using appropriate range checks. The data will be entered within one week of collection so that an up-to-date list of study participants is available to the study pediatricians for surveillance of outcome cases. Data files containing only the necessary variables for identification of study participants will be created for both the hospital surveillance and for questions from clinic nurses. Individual data entry clerks will be assigned specific clinics whose data they will enter and to whom they will send a weekly listing of those patients, initially randomized at their clinic, who were vaccinated the previous week at any study site; the study nurses will also be given a list of those patients who failed to return for scheduled visits. They will also be responsible for contacting the study nurses to resolve data queries.

Data from each data entry clerk will be backed up daily; the master file containing the data from all of the clinics will be updated and backed up weekly.

**XII. Study monitoring**

Monitoring will be the responsibility of the SAIMR investigators and WLVP and their local representatives. Independent local monitors hired by WLVP will perform primary monitoring of the study sites. Safety monitoring will be the responsibility of the Safety Monitoring Committee.

**A. Safety**

Vaccine safety will be monitored and reported as described in Section XI. Safety will be analyzed as the trial progresses, with reports on adverse reactions and outcome cases provided to the Safety Monitoring Committee on a quarterly basis. Deaths, life-threatening events, and unexpected events judged to be vaccine-related will be reported to the Committee monthly. All serious adverse events will be investigated.

**B. Progress of the Study**

A summary of the progress of the trial will be provided by the investigators to WLVP on a monthly basis and to the International Advisory Committee and the Safety Monitoring Committee on a quarterly basis.

**C. Verification of Data**

Study investigators from SAIMR and WLVP monitors will monitor the participating clinics. Monitoring visits will occur before the study begins, at regular intervals during the trial, and at the completion of the trial. Monitoring will be conducted to:

1. Assure that the Informed Consents are properly signed and dated. It is anticipated that a random sample of 20-25% of informed consents will be monitored.

2. Assure adherence to the protocol.

3. Audit and review data derived from and pertinent to the study for accuracy and completeness of information. This includes Vaccination Report forms, subject medical records, laboratory reports, etc. Approximately 20-25% of report forms will be randomly monitored.

4. Personnel from SAIMR will examine records for accurate documentation of quantity and date of receipt of investigational vaccine, dispensation and accountability data regarding administration to each study participant, loss of investigational materials, and unused supplies.

5. Review Investigator files for required documents, e.g. IRB protocol approvals, protocol amendments, and informed consents.

6. Prepare monitoring reports that record and report observations on the progress of the trial, continued acceptability of the facilities, and fulfillment of obligations by the Investigator.

**XIII. Study advisory committees**

Two independent advisory committees have been established to assist in the design, on-going implementation, and interpretation of study results.

**A. International Advisory Committee (IAC)**

An International Advisory Committee has been established whose role is as an advisory body to make recommendations to the Principal Investigator and the manufacturer.

The specific roles/responsibilities of the committee are as follows:

1. The IAC will provide technical advice and oversight on protocol development; on data collection, management, and analysis; and on ways to improve the conduct of the trial, if any are identified, during the trial. The IAC will review data and advice from the Safety Monitoring Committee, progress reports from the Principal Investigator, and reports from any site visitors or consultants. The IAC will have the authority to recommend the trial be stopped, if it concludes, on the basis of information provided to it, that the vaccine is unsafe or that for other reasons it would be unethical to continue. The IAC may also recommend the trial be extended, if additional enrollment and surveillance would increase the statistical power of the study.

2. Membership of the IAC will consist of individuals, none of whom have any formal connection with the vaccine manufacturer WLVP. Individuals for the Committee were chosen based on their expertise in the field of acute respiratory infections in children or in the conduct of clinical trials such as the one proposed. The Committee will comprise:

Dr. Claire Broome

US Centers for Disease Control and Prevention

Dr. Nate Pierce (Chairman)

Division of Child Health and Development

The Johns Hopkins University

Dr. Kim Mulholland

ARI Programme

World Health Organization

Dr. N. E. Khomo

District Health Care Services

City Health Department

Greater Germiston Transitional Metropolitan Council

Dr. William Blackwelder

Biometry Section

US National Institute of Allergy and Infectious Diseases

3. The Principal Investigator will actively participate in IAC meetings, while participants from WLVP will have observer status. The Committee also has the option of holding some meetings in closed session. Both the Principal Investigator and WLVP participants will receive copies of all Committee documents.

4. The World Health Organization will provide support and serve as a focal point for communication among the committees, WLVP, and the Principal Investigator. The secretariat will provide summaries of the discussions and recommendations of each IAC and Safety Monitoring Committee meeting and will coordinate the planning for these meetings.

5. The IAC will meet at least once a year or more frequently, if requested by the Chairperson, to review the trial progress and consider information or advice provided by the Safety Monitoring Committee. The IAC and the Safety Monitoring Committee should schedule some meetings concurrently to facilitate interaction and discussion between the two committees.

**B. Safety Monitoring Committee (SMC)**

Safety monitoring will be the primary responsibility of the Committee. The SMC will monitor for severe, long-term effects of vaccination, assess the vaccine risk in HIV-infected children, and assess the overall risk of vaccine failure. The specific responsibilities of the Committee will be defined at the first meeting of the Committee prior to initiation of the Phase III trial. An amendment to the protocol will be filed subsequently defining their responsibilities. At the conclusion of the study, the SMC will review the complete safety data and write an independent report summarizing the findings of the study. This report will be submitted to the Principal Investigator and WLVP.

1. The individual members of the committee, who do not participate in study management or data classification decisions, are:

Professor P. Cooper

Department of Pediatrics

Johannesburg Hospital

Dr. Rob Breiman

US National Vaccine Program

Professor S.S. Abdool Karim

Center for Epidemiological Research in Southern Africa

Medical Research Council

Durban, South Africa

Dr. R. Borok, biostatistician from SAIMR, will serve as statistical advisor to the committee.

2. The SMC’s primary responsibility is for safety monitoring and to maintain the vaccine code. The monitors can break the code to analyze safety data, when it is considered appropriate. Prior to initiation of the trial the SMC members will establish general criteria regarding deaths, and unexpected serious adverse events or unacceptable rates of adverse events which would recommend premature termination of the trial.

3. Reports related to safety issues will be reviewed and analyzed at regular intervals to be defined by the SMC members. The SMC is expected to inform the IAC on issues that interfere with the conduct of the trial, including any intended breaking of the code or recommendations on the continuation or interruption of the trial due to safety issues.

4. In order to keep IAC members blinded, the SMC in some instances may be asked to review data unrelated to safety during the trial and to make recommendations to the IAC based on this review.

**XIV. Rules for unblinding the study**

The randomization code will be broken:

A. For reasons of safety or excess mortality, as determined by the Safety Monitoring Committee. In the event of a serious adverse reaction following vaccination, the code may be broken for individual children. The identity of the randomization group may be provided to the child’s parents, physician, and to the study Investigator, if deemed necessary by the Safety Monitoring Committee. The Committee also has the authority to unblind the trial and recommend to the IAC that the trial be terminated should safety issues warrant such action.

B. For reasons of lack of vaccine efficacy. Outcome cases of pneumococcal disease will be unblinded and reviewed quarterly by the Safety Monitoring Committee. The Committee will be provided with estimates of the number of cases in each vaccine group that would be required to consider the vaccine without efficacy. The Safety Monitoring Committee may recommend to the International Advisory Committee that the trial be terminated, based on their findings.

C. Upon completion of enrollment and follow-up of study participants and termination of the trial.

**XV. Target dates**

Projected initiation of trial: January, 1998

Projected completion of subject enrollment: December, 1999

Projected completion of patient follow-up/termination of the study: January, 2001

**XVI. Study management**

**A. Monitoring**

A representative of WLVP will be responsible for establishing the schedule and procedures to be followed for monitoring this study. Prior to the beginning of the study, the Investigator will be informed as to the anticipated frequency of the monitoring visit at each study site. In addition, the Investigator will receive reasonable notification prior to each monitoring visit during the course of the study.

**B. Protocol Revisions**

With the exception of emergency situations, no changes or deviations in this protocol will be permitted without the documented approval of a representative of WLVP and the Institutional Review Boards that granted original approval for the study. This stipulation does not apply to those changes made necessarily to reduce discomfort or overt risk to subjects. In the event of any emergency, the Investigator shall institute any medical procedures, which he/she deems appropriate. However, all such events and procedures must be promptly reported to WLVP.

**C. Data management**

Primary data management of all data forms will be done locally. The forms will then be reviewed at WLVP.

**D. Publications**

WLVP reserves the right to review and comment upon any manuscripts intended for publication or public presentation which encompass information obtained during clinical studies sponsored or supported by WLVP prior to its submission for presentation.

**E. Materials**

Materials (defined as vaccines and subject sera) are the property of WLVP and cannot be used by the Investigator for their own experimental purposes without written permission from WLVP.

**F. Indemnification**

A standard indemnification agreement between the Principal Investigator and American Cyanamid Company will be supplied.

**XVII. References**

1. World Health Organization. Programme for the Control of Acute Respiratory Tract Infections. Pneumococcal conjugate vaccines. Report of a meeting, Geneva, 15-17 November 1993. WHO\ARI\94.34.

2. Childrens Vaccine Initiative. CVI Forum. A pneumococcal vaccine to save children of all ages nears final testing. 1996; **13**:3-12.

3. Forgie IM, O’Neill KP, Lloyd-Evans N, Leinonen M, et al. Etiology of acute lower respiratory tract infections in Gambian children:ALRI in infants presenting at the hospital. Paed. Infect. Dis. J. 1991; **10**:33-41.

4. Forgie IM, O,Neill KP, Lloyd-Evans N, Leinonen M, et al. Etiology of acute lower respiratory tract infections in Gambian children:ALRI in children ages one to nine years presenting at the hospital. Pediat. Infect. Dis. J. 1991; **10**:42-47.

5. Mulholland K, Hilton S, Adegbola R, Usen s, et al. Randomised trial of *Haemophilus influenzae* type-b tetanus protein conjugate for prevention of pneumonia and meningitis in Gambian infants. Lancet 1997; **349**:1191-1197.

6. Davidson M, Schraer CD, Parkinson AJ, et al. Invasive pneumococcal disease in an Alaska native population, 1980 through 1986. JAMA 1989; **261**:715-718.

7. O’Dempsey TJ, Mcardle TF, Lloyd-Evans N, et al. Pneumococcal disease among children in a rural area of West Africa. Paed. Infect. Dis. J. 1996; **15**:431-437.

8. Zangwill K, Vadeheim CM, Vannier AM, et al. Epidemiology of invasive pneumococcal disease in Southern California: Implications for the design and conduct of a pneumococcal conjugate vaccine efficacy trial. J. Infect. Dis. 1997; **174**: 752-889.

9. Eskola J, Takala AK, Kela E, et al. Epidemiology of invasive pneumococcal disease in children in Finland. JAMA 1992; **268**:3323-3327.

10. Burman L, Norrby R, Trollfors B. Invasive pneumococcal disease: Incidence, predisposing factors, and prognosis. Rev. Infect. Dis. 1985; **7**:133-142.

11. Dagan R, Engelhard D, Piccar E, et al. Epidemiology of invasive childhood pneumococcal infection in Israel. JAMA 1992; **268**:3328-3332.

12. Crewe-Brown HH, Karstaedt AS, Saunders GL, et al. *Streptococcus pneumoniae* bacteremia and HIV infection: Alteration in penicillin susceptibility and serogroups/serotypes. Clin Infect. Dis. 1997, in press

13. Daponte BO. Results from the 1995 Soweto population survey: Estimates of the population of towns in Soweto. 1995, Human Rights Institute of South Africa.

14. Friedland IR. Comparison of the response to antimicrobial therapy of penicillin-resistant and penicillin-susceptible pneumococcal disease. Paed. Infect. Dis. J. 1995; **14**:885-890.

15. Friedland IR, Klugman KP. Antibiotic-resistant pneumococcal disease in South African children. AJDC 1992; **146**920-923.

16. Garenne M, Ronsmans C, Campbell H. The magnitude of mortality from acute respiratory infection in children under 5 years in developing countries. Wld. Hlth. Stats. Quart. 1992; **45**:180-191.

17. Progress towards health for all: Third monitoring report: Resolution of the World Health Assembly 34.36. Wld. Hlth. Statis. Quart. 1995; **48**:174-199.

18. UNICEF. The State of the Worlds Children 1996. Statistical Tables. 1996, pg 90-98.

19. Von Schirnding YER, Yach D, Klein M. Acute respiratory infection as an important cause of childhood deaths in South Africa. SAMJ. July 1991; **80**:79-82.

20. Nathoo KJ, Nkrumah FK, Ndlovu D, et al. Acute lower respiratory tract infections in hospitalized children in Zimbabwe. Ann. Trop. Paed. 1993; **13**:253-261.

21. Mao C, Harper M, McIntosh K, et al. Invasive pneumococcal infection in human immunodeficiency virus infected children. J. Infect. Dis. 1996; **173**: 870-875.

22. Ahmed F, Steinhoff MC, Rodriquez-Barradas MC, et al. Effect of human immunodeficiency virus type 1 infection on the antibody response to a glycoprotein conjugate pneumococcal vaccine: Results from a randomized trial. J. Infect. Dis.1996; **173**:83-90.

23. Makela PH, Luotenen J, Pukander J et al. Pneumococcal vaccine and otitis media. Lancet. September 1980;547-551.

24. Douglas RM, Paton JC, Duncan SJ, Hansman DJ. Antibody response to pneumococcal vaccination in children younger than 5 years of age. J. Infect. Dis. 1983; **148**:131-137.

25. Temple K, Greenwood P, Inskip H, Hall A, Koskela M, Leinonen M. Antibody response to pneumococcal capsular polysaccharide vaccine in African children. Paed.Infect. Dis. 1991; **10**:386-390.

26. Adams G, Deaverk A, Cochi SL, et al. Decline of childhood *H. influenzae* type b (Hib) disease in the Hib vaccine era. JAMA. 1993; **269**: 221-225.

27. Takala AK, Eskola J, Leinonen M, Kahthy H, et al. Reduction of oropharyngeal carriage of  *Haemophilus influenzae* type b (Hib) in children immunized with an Hib conjugate vaccine. J. Infect. Dis. 1991; **164**:982-986.

28. Black SB, Shinefield HR, Kaiser Permanente Pediatric Vaccine study group. Immunization with oligosaccharide conjugate *H. influenzae* (HbOC) vaccine on a large health maintenance organization population: Extended follow-up and impact on *H. influenzae* disease epidemiology. Paed. Infect. Dis. J. 1992; **11**: 610-613.

29. Black SB, Shinefield HR, Fireman B, et al. Efficacy in infancy of oligosaccharide conjugate *Haemophilus influenzae* (HbOC) vaccine in a United States population of 61,080 children. Paed. Infect. Dis. J. 1991;**10**:97-104.

30. Mohle-Boetani JC, Ajello G, Breneman E, et al. Carriage of *Haemophilus influenzae* type b in children after widespread vaccination with conjugate Hib vaccines. Paed. Infect. Dis. J. 1993; **12**:593-599.

31. Murphy TD, Pastor P, Medley F, et al. Decreased Haemophilus colonization in children vaccinated with Hib conjugate vaccine. J Pediatr 1993;**122**:517-523.

32. Makala AK, Santosham M, Almeido-Hill J, et al. Vaccination with Hib-meningococcal protein conjugate vaccine reduces oropharyngeal carriage of Hib among American Indian children. Paed. Infect. Dis. J. 1993; **12**: 593-599.

33. Anderson EL, Kenned DJ, Geldmacher KM, Donnely J, Mendelman PM. Immunogenicity of heptavalent pneumococcal conjugate vaccine in infants. J. of Pediatrics. 1996; **128**:649-653.

34. Kathy H, Ahman H, Ronnberg PR, Tillkainen R, Eskola J. Pneumococcal polysaccharide-meningococcal outer membrane protein complex conjugate vaccine is immunogenic in infants and children. J. Infect. Dis. 1995; **172**: 1272-1278.

35. Sniadack DH, Schwartz B, Lipman H, et al. Potential interventions for the prevention of childhood pneumonia: Geographic and temporal differences in serotype and serogroup distribution of sterile site pneumococcal isolates from children- Implications for vaccine strategies. Paed. Infect. Dis. J. 1995; **14**:503-510.

36. Klugman KP, Koornhof HJ. Drug resistance patterns and serogroups or serotypes of pneumococcal isolates from cerebrospinal fluid or blood, 1979-1986. J. Infect. Dis.1988;**158**: 956-964.

**Appendix 1:**  **Data of most common infection-related diagnosis in 1995 compared to 1993 and 1994. ^1^**

| **Primary diagnosis** | **1993** | **1994** | **1995** | **% of total admissions’95 ^2^** | **Related deaths-’95^3^** |
| --- | --- | --- | --- | --- | --- |
| **Bronchopneumonia** | 883 | 1156 | 1478 | 30.5 | 93 |
| **Bronchiolitis** | 148 | 166 | 204 | 4.2 | 0 |
| **Pulmonary TB** | 96 | 135 | 140 | 2.9 | 1 |
| **TB meningitis** | 14 | 17 | 9 | 0.2 | 3 |
| **Bacterial meningitis** | 132 | 120 | 122 | 2.5 | 15 |
| **Septicemia** | 228 | 274 | 301 | 6.2 | 53 |
| **Urinary tract infection** | 147 | 129 | 156 | 3.2 | 0 |
| **HIV infection- New** | 135 | 160 | 286 | 8.6 ^4^ | 86 ^5^ |
| **HIV infection- Old** | 44 | 67 | 130 |  |  |
| **Gastro-enteritis** | 733 | 771 | 937 | 15.9 | 48 |

^1^ Data were derived from the Chris Hani Baragwanath Hospital ward –stats file, courtesy of Dr. U Kala.

^2^ Percentages are calculated on the basis of all ward admissions.

^3^ These are deaths recorded in 1995, due to the attached diagnosis.

^4\5^ Figures recorded are totals for new and old cases of HIV diagnosis combined.

**Appendix 2:**

**Appendix 3: Immunization clinics in Soweto and Orange Farm**

**Soweto**

Chiawelo and Kliptown mobile clinic

Jabavu

Mofolo South

Orlando and mobile clinic

Pimville

Shanty

Senaoane

Tladi and 2 mobile clinics

Zola and mobile clinic

Zondi

Dobsonville

Meadowlands

Doornkop and 2 satellite clinics

**Orange Farm**

Stetford and 3 satellite clinics

**Appendix 4a:** **Subject information sheet for study participation**

Pneumonia and meningitis (an infection of the surroundings of the brain) are serious diseases in young children. These diseases can be caused by a germ called *Streptococcus pneumoniae* (pneumococcus) or by another germ called *Haemophilus influenzae* type b (haemophilus).

We, the doctors and nurses of the SAIMR vaccine study, together with the Department of Health for Soweto, the World Health Organization, and the vaccine manufacturing company, Wyeth Lederle Vaccines and Pediatrics, are trying to find a vaccine that will prevent these diseases. A vaccine is available to prevent diseases caused by haemophilus. This vaccine is routinely available in the US and is now available in SA. Children in Soweto do not routinely receive this vaccine as it is very expensive. A new vaccine is being studied that may prevent diseases caused by the pneumococcus. We would like to test if the vaccine can prevent diseases caused by the pneumococcus in young children in Soweto. All of the 44,000 children born in Soweto in 1998 and 1999 will be able to take part in this study.

Participation in the study is voluntary, however, if you agree to have your child take part in the study, then your child will receive his/her routine vaccinations and the haemophilus vaccine. He/she will also receive either the pneumococcus trial vaccine or a placebo, which is an inactive solution. Half of the children in the study will receive the pneumococcus vaccine and the other half will receive the placebo. Whether your child receives the study vaccine or placebo will be determined randomly. The trial vaccine and placebo will be given at the same time that your child receives the other routine vaccines at 6, 10 and 14 weeks of age.

If your child takes part in the study, he/she will be protected from the pneumonia and meningitis caused by the haemophilus bacteria. Your child may also be protected from disease caused by the pneumococcus bacteria. In order for your child to receive these vaccines, you will have to agree to have your child take part in the study. In 1997 we injected 500 children with the vaccine and found that some children may have a slight fever after vaccination or a lump or redness at the vaccination site but these were not serious and went away without treatment.

If your child is admitted to the hospital, a small amount of blood (2 ml = ½ teaspoon) will be taken for diagnostic tests for pneumonia. There may be a small bruise after the blood is taken but again this should not be serious.

If your child is to be in the study, you must also agree, if possible, to bring your child to the clinic if your child is ill at any time during the study. If your child develops a reaction after vaccination which concerns you, you should also contact either the clinic nurse or one of the study doctors. Any additional medical care required as a result from injury by the vaccine/biological furnished by Wyeth-Lederle Vaccines and Pediatrics for use in this clinical study, provided that such injury was incurred without the investigator's negligence or intentional acts of misconduct, will be provided at the clinic. As required by the Association of the British Pharmaceutical Industry, if there are costs to you for such injuries as a result of your child's participation in this study, those costs will be covered by Wyeth Lederle Vaccines and Pediatrics. If you do not wish your child to be in the study, your child will receive the routine vaccines and will receive the standard care at the clinic. If you wish to take your child out of the study, you may do so at any time, and your child will also receive the usual vaccines and will not be less well treated at the clinic.

All information about you and your child in this study is confidential but must be available for inspection to SAIMR investigators, to the sponsor, Wyeth Lederle Vaccines and Pediatrics, and to the US Food and Drug Administration (FDA) or other government regulators. At the end of the study we will discuss the results of your child's vaccination with you. If you have questions, are not treated fairly or believe you have been hurt by the study, contact us at the clinic.

If your child is ill during the study you may come to the clinic or call Dr. Mbelle at 934 - 2729, Dr. Madhi at 942-3559, or Professor K P Klugman at 489 - 9010

Signature - Parent/Guardian

Date

Signature - Witness

Date

**Appendix 4b:** **Subject information sheet for immunogenicity study**

Your child has been taking part in a study on a new vaccine to prevent pneumonia and meningitis caused by a germ called pneumococcus. To be certain that the vaccine is producing a good immune response, we need to take blood from 200 children at four weeks after their last dose of the vaccine and measure the protection that the child has received from the vaccine. Your child has been randomly chosen to have his/her blood tested for protection against the pneumococcus germ. If you agree, we will take about ½ teaspoon of blood from your child. There may be a small bruise after the blood is taken but this should not be serious.

As required by the Association of the British Pharmaceutical Industry, if there are costs to you for such injuries as a result of your child's participation in this study, those costs will be covered by Wyeth Lederle Vaccines and Pediatrics. If you do not wish your child to have blood taken, your child will continue to be in the vaccine study and will receive the same care as the other children in the study.

All information about you and your child in this study is confidential but must be available for inspection to SAIMR investigators, to the sponsor, Wyeth Lederle Vaccines and Pediatrics, and to the US Food and Drug Administration (FDA) or other government regulators. At the end of the study we will discuss the results of your child's vaccination with you. If you have questions, are not treated fairly or feel you have been hurt by the study, contact us at the clinic.

If your child is ill during the study or you have any questions you may come to the clinic or call Dr. Mbelle at 934 - 2729, Dr. Madhi at 942-3559, or Professor K P Klugman at 489 - 9010

Signature - Parent/Guardian

Date

Signature - Witness

Date
