# Supplementary material for: Use of Procalcitonin and C-Reactive Protein to Evaluate Vaccine Efficacy against Pneumonia
Source: PLoS Med. 2005 Feb 22;2(2):e38. doi: 10.1371/journal.pmed.0020038 (PMC549587; doi:10.1371/journal.pmed.0020038)
Supplement: Protocol S2 — (378 KB DOC). [file pmed.0020038.sd002.doc]

Human Research Ethics Committee (Medical)
(formerly Committee for Research on Human Subjects (Medical)


University of the Witwatersrand, Johannesburg
Secretariat: Research Office, Room SH10005, 10th floor, Senate House - Telephone: +27 11 717-1234 - Fax: +27 11 339-5708 Private Bag 3, Wits 2050, South Africa

PC-J378dskl2/es
23 September 2004
TO WHOM IT MAY CONCERN AT PLOS MEDICINE
I confirm that projects
M03-09-22
M970196 -
To determine the potential role of procalcitonin in better defining the efficacy of a nonavalent pneumococcal conjugate vaccine
Double blind, randomised trial of a nonavalent pneumococcal conjugate vaccine to reduce the incidence of invasive pneumococcal disease and pneumonia requiring hospitalisation in infants
were reviewed and approved by our ethics committee

Professor Peter Cleaton-Jones
BDS, MBBCH, PhD, DSc (Dent), DTM&H, DPH, DA, MASSAF. Chair, Human Research Ethics Committee (Medical)
