# Supplementary material for: Use of Procalcitonin and C-Reactive Protein to Evaluate Vaccine Efficacy against Pneumonia
Source: PLoS Med. 2005 Feb 22;2(2):e38. doi: 10.1371/journal.pmed.0020038 (PMC549587; doi:10.1371/journal.pmed.0020038)
Supplement: Protocol S3 — (24 KB DOC). [file pmed.0020038.sd003.doc]

**Appendix 4a:** **Subject information sheet for study participation**

Pneumonia and meningitis (an infection of the surroundings of the brain) are serious diseases in young children. These diseases can be caused by a germ called *Streptococcus pneumoniae* (pneumococcus) or by another germ called *Haemophilus influenzae* type b (haemophilus).

We, the doctors and nurses of the SAIMR vaccine study, together with the Department of Health for Soweto, the World Health Organization, and the vaccine manufacturing company, Wyeth Lederle Vaccines and Pediatrics, are trying to find a vaccine that will prevent these diseases. A vaccine is available to prevent diseases caused by haemophilus. This vaccine is routinely available in the US and is now available in SA. Children in Soweto do not routinely receive this vaccine as it is very expensive. A new vaccine is being studied that may prevent diseases caused by the pneumococcus. We would like to test if the vaccine can prevent diseases caused by the pneumococcus in young children in Soweto. All of the 44,000 children born in Soweto in 1998 and 1999 will be able to take part in this study.

Participation in the study is voluntary, however, if you agree to have your child take part in the study, then your child will receive his/her routine vaccinations and the haemophilus vaccine. He/she will also receive either the pneumococcus trial vaccine or a placebo, which is an inactive solution. Half of the children in the study will receive the pneumococcus vaccine and the other half will receive the placebo. Whether your child receives the study vaccine or placebo will be determined randomly. The trial vaccine and placebo will be given at the same time that your child receives the other routine vaccines at 6, 10 and 14 weeks of age.

If your child takes part in the study, he/she will be protected from the pneumonia and meningitis caused by the haemophilus bacteria. Your child may also be protected from disease caused by the pneumococcus bacteria. In order for your child to receive these vaccines, you will have to agree to have your child take part in the study. In 1997 we injected 500 children with the vaccine and found that some children may have a slight fever after vaccination or a lump or redness at the vaccination site but these were not serious and went away without treatment.

If your child is admitted to the hospital, a small amount of blood (2 ml = ½ teaspoon) will be taken for diagnostic tests for pneumonia. There may be a small bruise after the blood is taken but again this should not be serious.

If your child is to be in the study, you must also agree, if possible, to bring your child to the clinic if your child is ill at any time during the study. If your child develops a reaction after vaccination which concerns you, you should also contact either the clinic nurse or one of the study doctors. Any additional medical care required as a result from injury by the vaccine/biological furnished by Wyeth-Lederle Vaccines and Pediatrics for use in this clinical study, provided that such injury was incurred without the investigator's negligence or intentional acts of misconduct, will be provided at the clinic. As required by the Association of the British Pharmaceutical Industry, if there are costs to you for such injuries as a result of your child's participation in this study, those costs will be covered by Wyeth Lederle Vaccines and Pediatrics. If you do not wish your child to be in the study, your child will receive the routine vaccines and will receive the standard care at the clinic. If you wish to take your child out of the study, you may do so at any time, and your child will also receive the usual vaccines and will not be less well treated at the clinic.

All information about you and your child in this study is confidential but must be available for inspection to SAIMR investigators, to the sponsor, Wyeth Lederle Vaccines and Pediatrics, and to the US Food and Drug Administration (FDA) or other government regulators. At the end of the study we will discuss the results of your child's vaccination with you. If you have questions, are not treated fairly or believe you have been hurt by the study, contact us at the clinic.

If your child is ill during the study you may come to the clinic or call Dr. Mbelle at 934 - 2729, Dr. Madhi at 942-3559, or Professor K P Klugman at 489 - 9010

Signature - Parent/Guardian

Date

Signature - Witness

Date
